# Closed-loop cycles of experiment design, execution, and learning accelerate systems biology model development in yeast

**DOI:** 10.1073/pnas.1900548116

**Published:** 2019-08-16

**Authors:** Anthony Coutant, Katherine Roper, Daniel Trejo-Banos, Dominique Bouthinon, Martin Carpenter, Jacek Grzebyta, Guillaume Santini, Henry Soldano, Mohamed Elati, Jan Ramon, Celine Rouveirol, Larisa N. Soldatova, Ross D. King

**Affiliations:** ^a^Le Laboratoire d’Informatique de Paris-Nord (LIPN), UMR CNRS 7030, University Paris 13, F-93430 Villetaneuse, France;; ^b^Manchester Institute of Biotechnology, University of Manchester, M1 7DN Manchester, United Kingdom;; ^c^School of Computer Science, University of Manchester, M13 9PL Manchester, United Kingdom;; ^d^Institute of Systems and Synthetic Biology (iSSB), CNRS UMR8030, University Paris-Saclay, Genopole, 91030 Evry, France;; ^e^Department of Computer Science, University of Brunel, UB8 3PH London, United Kingdom;; ^f^Muséum National d’Histoire Naturelle, L’Institut de Systématique, Évolution, Biodiversité, UMR CNRS 7205, Sorbonne Université, 75005 Paris, France;; ^g^INSERM U908, Lille University, F-59655 Villeneuve d’Ascq, France;; ^h^Institut National de Recherche en sciences du numérique (INRIA), Lille Nord Europe, 59650 Lille, France;; ^i^Department of Computing, Goldsmiths, University of London, SE14 6NW London, United Kingdom;; ^j^Alan Turing Institute, NW1 2DB London, United Kingdom;; ^k^Artificial Intelligence Research Center, National Institute of Advanced Industrial Science and Technology, Koto, 135-0064 Tokyo, Japan

**Keywords:** artificial intelligence, machine learning, diauxic shift

## Abstract

One of the most challenging tasks in modern science is the development of systems biology models: Existing models are often very complex but generally have low predictive performance. The construction of high-fidelity models will require hundreds/thousands of cycles of model improvement, yet few current systems biology research studies complete even a single cycle. We combined multiple software tools with integrated laboratory robotics to execute three cycles of model improvement of the prototypical eukaryotic cellular transformation, the yeast (*Saccharomyces cerevisiae*) diauxic shift. In the first cycle, a model outperforming the best previous diauxic shift model was developed using bioinformatic and systems biology tools. In the second cycle, the model was further improved using automatically planned experiments. In the third cycle, hypothesis-led experiments improved the model to a greater extent than achieved using high-throughput experiments. All of the experiments were formalized and communicated to a cloud laboratory automation system (Eve) for automatic execution, and the results stored on the semantic web for reuse. The final model adds a substantial amount of knowledge about the yeast diauxic shift: 92 genes (+45%), and 1,048 interactions (+147%). This knowledge is also relevant to understanding cancer, the immune system, and aging. We conclude that systems biology software tools can be combined and integrated with laboratory robots in closed-loop cycles.

## Significance

Systems biology involves the development of large computational models of biological systems. The radical improvement of systems biology models will necessarily involve the automation of model improvement cycles. We present here a general approach to automating systems biology model improvement. Humans are eukaryotic organisms, and the yeast *Saccharomyces cerevisiae* is widely used in biology as a “model” for eukaryotic cells. The yeast diauxic shift is the most studied cellular transformation. We combined multiple software tools with integrated laboratory robotics to execute three semiautomated cycles of diauxic shift model improvement. All the experiments were formalized and communicated to a cloud laboratory automation system (Eve) for execution. The resulting improved model is relevant to understanding cancer, the immune system, and aging.

Systems biology presents an extreme challenge to the traditional human-based scientific method (1, 2). The fundamental difficulty is the high degree of complexity of biological systems, where even simple “model” systems such as *Escherichia coli* and *Saccharomyces cerevisiae* have thousands of genes, proteins, and small molecules all interacting together in complicated spatial-temporal ways. This biological complexity implies a need for a similar complexity, probably beyond human intuitive understanding, in the corresponding systems biology models.

In the development of systems biology models, biological knowledge is integrated to form a model, experiments are planned and executed to test the model, the experimental results are used to refine the model, new biological knowledge is generated, and the cycle repeated (1). To radically improve existing system biology models, it will be necessary to execute hundreds/thousands of such cycles of model improvement. However, little current research completes even a single cycle. We therefore argue that greater automation is required, which will in turn require the combination and integration of multiple systems biology software tools into closed-loop cycles with laboratory robotics.

To evaluate the integration of software tools and laboratory robotics for systems biology we selected as a test case the diauxic shift of the yeast *S. cerevisiae.* This is the standard model system for understanding eukaryotic cellular transformation, and it is relevant to understanding cancer (Warburg effect), the immune system, and aging. In *S. cerevisiae* growing in batch culture on glucose with aeration a diauxic shift is commonly observed: During the first growth phase, yeast metabolizes glucose using the fermentative Embden–Meyerhof pathway to produce ethanol (3); when the glucose is exhausted, it switches to a fully respiratory metabolism utilizing the tricarboxylic acid cycle and oxidative phosphorylation in the mitochondria (3). This transition requires the large-scale remodeling of the metabolic apparatus (4). However, despite being one of the most studied of all eukaryotic cellular transformations, the diauxic shift is still very poorly understood, and existing systems biology models of this transformation could be greatly improved.

**Fig. 1.**
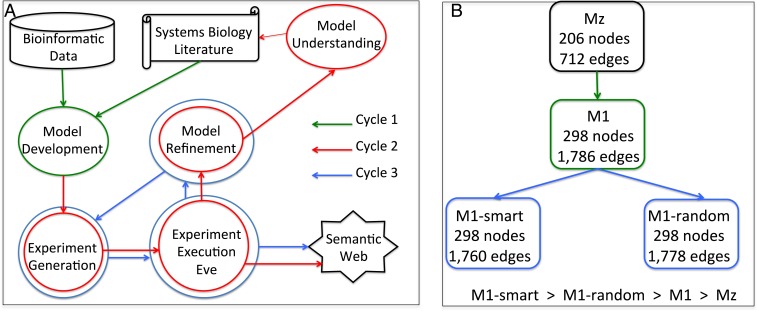
(*A*) In cycle 1 (green), the model M1 was developed by taking the model Mz from the literature and refining it based on bioinformatic data. In cycle 2 (red), the models M1-smart and M1-random were developed by running inference tools for experiment generation, experiment execution, and model refinement. The model M1-smart was analyzed for biological understanding. In cycle 3 (blue), the models M1-smart and M1-random were compared using experiment generation and experiment execution. (*B*) The relationship and details of systems biology models: Mz, M1, M1-smart, and M1-random.

**Fig. 2.**
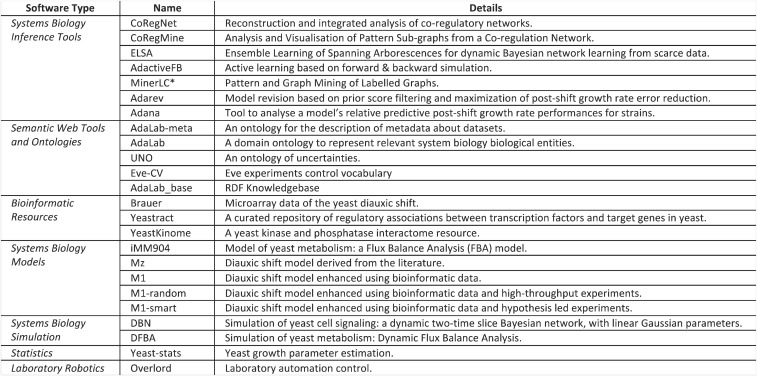
The implementation of closed-loop cycles in systems biology requires a wide range of different software: systems biology inference methods tools, semantic web tools and ontologies, bioinformatic resources, systems biology models, systems biology resources, statistical tools, and laboratory robotic systems.

We combined system biology software for data analysis, model formation, experiment generation, experiment execution, model refinement, systems biology modeling, bioinformatics, laboratory robotic control, and semantic web techniques to execute three cycles of diauxic shift model improvement (Fig. 1*A*). The wide range of software and tools required to achieve this are shown in Fig. 2. (CoRegNet and CoRegFlux are available in bioconductor. All of the other software is available on request at LIPN GitLab).

## Results

Modeling of the yeast diauxic shift is especially challenging because of the complexity of the biology involved, and the need to include subsystems operating at different time scales, and serving different purposes (5). The modeling requires integration of (1) a model of control of metabolism (cell signaling), and (2) a genome scale model of metabolism (1). The key difference between gene regulatory/signaling and metabolic networks is that the former carry signal flows, whereas metabolic pathways generate mass flows. We modeled the metabolic network as a biochemical (mechanistic) network based on the stoichiometry and reversibility of the reactions involved. Specifically, we chose the iMM904 model (6), (*SI Appendix 1*). This model is widely used, its structure is suitable for integration with signaling, and it is the most accurate model available for predicting growth phenotypes (7).

**Fig. 3.**
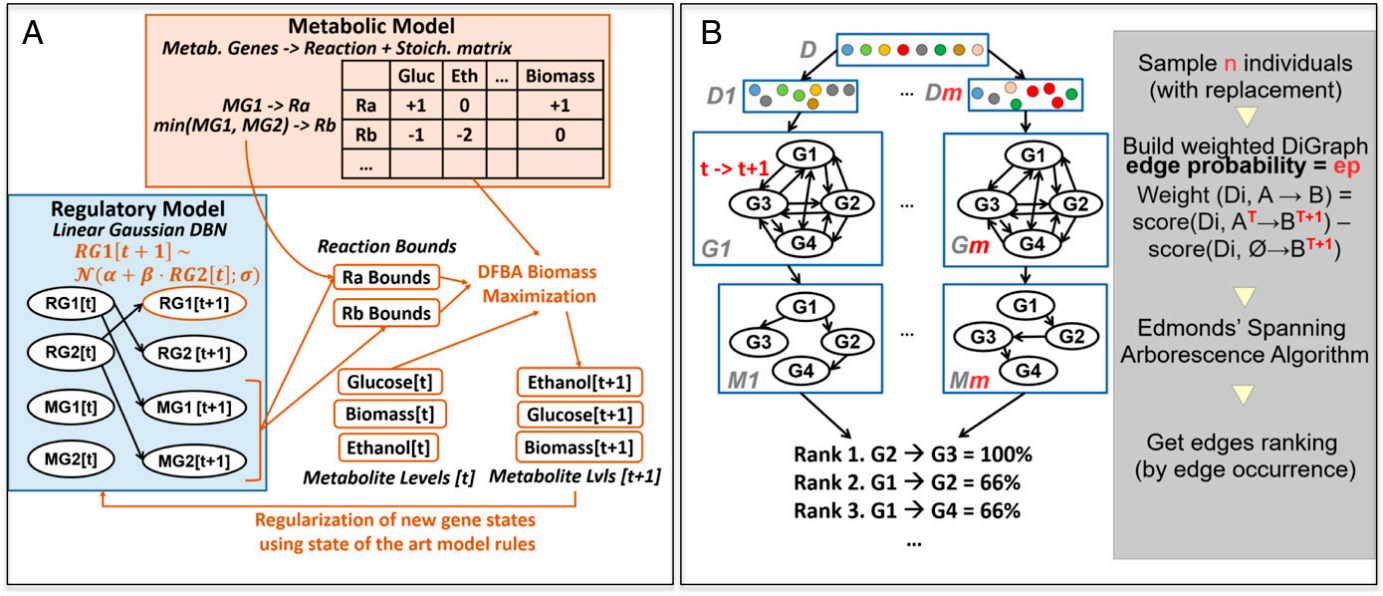
(*A*) The form of the integrated diauxic shift models. The regulatory model (blue box) is a DBN with linear Gaussian conditionals, overregulatory (parents and children) + metabolic (only parents) genes/proteins. The metabolic model (orange box) is composed of a stoichiometric matrix, and a set of enzymatic relations between metabolic genes and reactions. Simulation for *n* time steps consists of *n* repeats of: (1) DBN inference; (2) metabolic inference with dynamic flux balance analysis (DFBA); (3) regularization of gene states for the next time step using two results, and diauxic shift metabolite to gene rules. (*B*) The ensemble network inference procedure ELSA for learning DBNs. Simple models (“components”) are combined to form a consensual “composite” model. Each component is built by computing the Edmonds directed maximal spanning arborescence over a graph obtained by double sampling. The final composite model is built by aggregating all components by edge frequency to produce a ranking and postfiltering this using information from the Brauer dataset (4) (S5).

For cell-signaling modeling we used a two-time slice dynamic Bayesian network (DBN) with conditional linear Gaussian parameters (Fig. 3*A*). We selected this form of model because: it belongs to a well-studied family of continuous models, is easily interpretable in terms of activation and repression effects, and they enable the inference of gene states from known states in a versatile way. Each node in the model corresponds to either a regulatory protein or an enzyme, the former being the only type of node allowed to have children in the network, (*SI Appendix 2* and *3*). The starting point for our cell-signaling model was the model of Geistlinger et al. (8), which was assembled by compiling the findings of hundreds of scientific articles. We extracted the regulatory part of the model (Mz) and integrated this with iMM904m (Fig. 3*A*). Mz is of high quality in terms of dependencies recall (small number of false positive links), but it is relatively incomplete (missing links). Mz is also optimistic in that it predicts the occurrence of diauxic shifts for almost all strains with gene/protein deletions in the model.

In the first cycle of model improvement, the initial step was application of the bioinformatic program CoRegNet (9, 10) to identify genes potentially involved in control of the diauxic shift. CoRegNet integrates information from microarray experiments, regulatory interactions from the YEASTRACT database, and the *S. cerevisiae* Kinase and Phosphatase Interactome resource. CoRegNet uses a cooperative network based on shared transcription factor targets to identify coregulatory relationships from gene expression data (*SI Appendix 4*). We then applied a two-step model refinement process to its output (Fig. 3*B*): (*i*) We applied the ensemble network inference algorithm ELSA (Ensemble Learning of Spanning Arborescences) (11) to the Brauer microarray dataset (4), with Mz as a learning prior on the model space composed of the union of the Mz regulatory genes, the top 40 transcription factors identified by CoRegNet, and the top 40 kinases identified by CoRegNet (*SI Appendix 5*); (*ii*) We then applied a forward selection step to add to Mz edges that improve gene state predictions on the Brauer microarray dataset, using leave 1 out cross-validation. This generated model M1 (Fig. 1*B*).

**Fig. 4.**
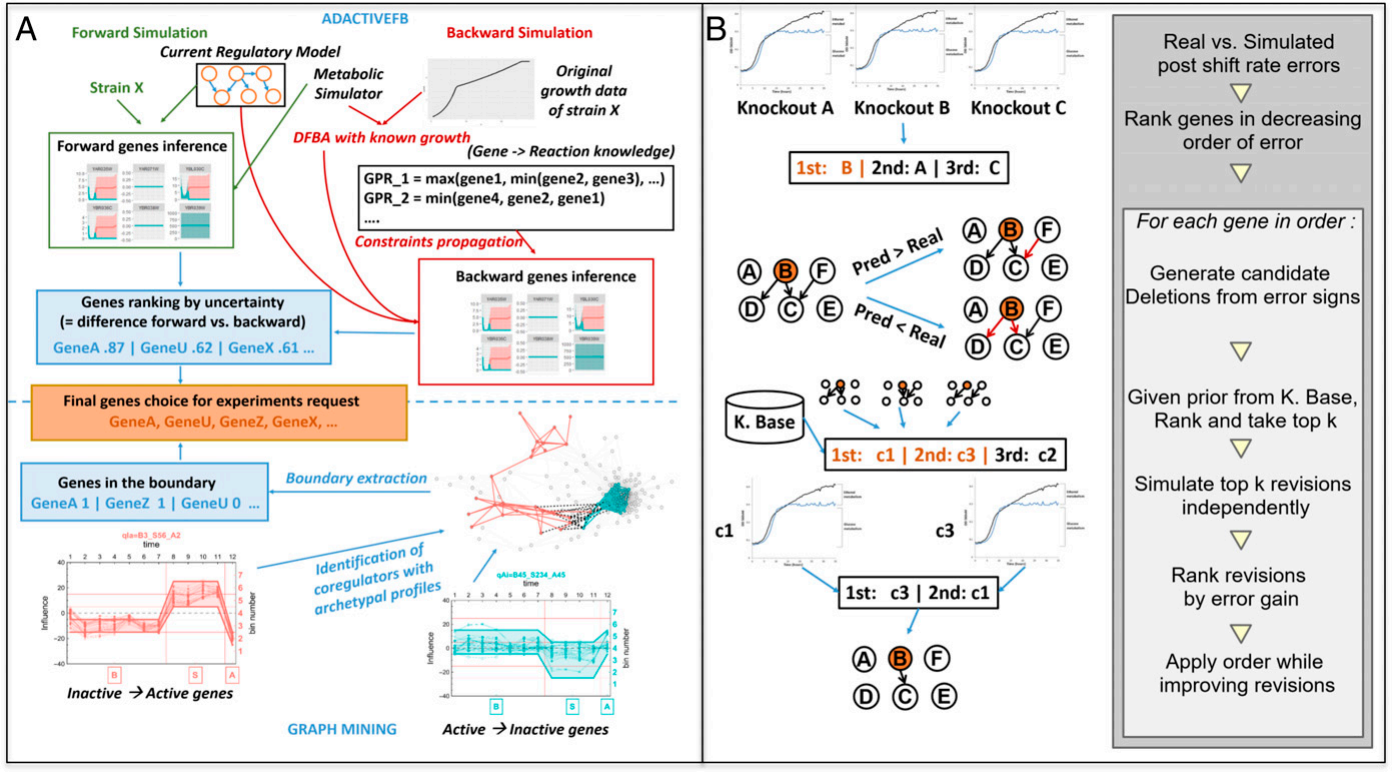
(*A*) The active learning tool AdactiveFB for selecting experiments identifies the most uncertain genes in a regulatory model using forward-backward simulation. It infers phenotype-driven distributions about genes in the regulatory model, which are then compared to gene distributions obtained from simulation. AdactiveFB first applies DFBA with fixed growth rates (instead of growth maximization) to estimate metabolic genes activity from growth curves by finding the metabolic reaction bounds associated to observed growth rates. It then propagates these constraints to the metabolic gene distributions in the regulatory model, before finally propagating them to regulatory genes using Bayesian inference. (*B*, *Left*) The tool CoRegMine used the Brauer dataset to form a graph of gene–target relationships. The graph mining tool MinerLC* selects genes belonging to a dense subgraph of the coregulation graph that have antagonist influence profiles along this time series, i.e., inactive → active vs. active → inactive denoted, respectively, by the red and blue nodes and links in the figure. Regulators at the border of those two subgraphs (i.e., nodes with active → inactive profiles which are neighbors—denoted by black links—of nodes with inactive → active profiles in the coregulation graph) are selected. (*B*, *Right*) The tool Adarev for model refinement. From the set of growth curves derived from for a set of knocked out genes, a set of prediction vs. observed postshift growth rate errors are computed and used to rank genes for removal. This ranking is used greedily to apply revisions starting with the most promising ones, iteratively validating the proposed changes as long as new predictions in the updated models are better than previous ones, in terms of postshift growth rate error reduction gain (S11).

At the start of the second cycle, we used tools to design experiments to provide the maximum amount of information to optimize the improvement of Mz to form M1, see Fig. 4*A*. We developed two tools for this task. The first tool is AdactiveFB (active learning based), which compares estimated protein/gene states (forward simulation) with the most likely protein/gene states consistent with the observed growth and metabolite state (backward simulation) (*SI Appendix 6*). In forward simulation a standard simulation from genes to phenotypes is performed, using both regulatory and metabolic simulators (*SI Appendix 3*). This produces an estimated time series of states for each gene in the DBN—as Gaussian distributions means and SDs. These forward simulations are compared with backward ones, i.e., simulations using phenotypes evidence to infer gene states (*SI Appendix 6*). Due to the unavailability of inferred states for several genes, the method used for backward simulation is designed to deal with partial evidence. The result of backward simulation is a set of backward time series for all of the genes in the regulatory model—also as Gaussian distributed means and SDs. Kullback–Leibler divergence is then calculated between the forward and backward Gaussian distributions (using their means and SDs) for each gene and each time point. This generates a divergence value for each (gene, time) pair. The genes selected for knockout experiment are those with the highest node divergence values. The strength of the AdactiveFB approach is that it focuses directly on optimizing the model rather than using a proxy. Its current main weakness is that the observed growth curve is the only phenotype used to inform backward simulation. Growth curve experiments are relatively robust (12), but they are not highly informative. In the future we plan to include many more phenotypic experiments.

The second tool, CoRegMine, initially uses CoRegNet (10) to infer a graph in which the vertices are coregulators labeled according to their influence profile (13), and the edges relate predicted coregulators. This graph is then processed by the graph mining tool MinerLC* (14) to extract subgraphs, each consisting of coregulators with similar influence profiles. Subgraph pairs were selected with a) antagonistic influence profiles, and b) edges relating coregulators from the two subgraphs, suggesting differential regulation of their common targets during the diauxic shift. The coregulators identified in this manner were then selected for use in experiments (*SI Appendix 7*). The strength of this approach is its use of background knowledge. Its weakness is that it does not directly focus on improving the model.

**Fig. 5.**
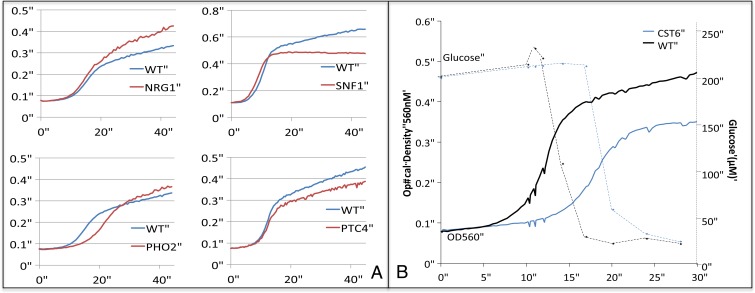
(*A*) Examples of diauxic shift phenotypes. The growth experiments executed by Eve revealed a wide variety of phenotypes: lower/faster growth rates (in fermentative/respiratory metabolism), and lower/greater growth yields. Each growth curve is made up of the mean OD_560_ readings for a strain (from a minimum of eight replicates) over 45 h, vs. wild type BY4741 (WT) with paired starting culture OD values. (*B*) An example of a glucose metabolism phenotype. Glucose consumption takes place most rapidly during the fermentative growth phase, with glucose levels generally depleted before the second period of slower growth.

We used the cloud laboratory robotics system Eve (15) to execute the experiments selected by AdactiveFB and CoRegMine. Eve executed two complementary types of automated experiment on selected yeast deletion strains: determinations of growth curves (Fig. 5*A*) and glucose consumption curves (Fig. 5*B*). The observed growth curves were preprocessed, normalized, and descriptive parameters calculated (12) (*SI Appendix 8*). Periodic colorimetric resorafin-based assays were used to track glucose levels in the culture medium.

The next step in the cycle of model improvement is to refine the model based on the obtained new experimental results. We developed the tool Adarev for this task. As Eve’s experiments do not directly observe the time-series of protein/gene states, these need to be inferred from observations of growth and limited metabolite states. The approach used by Adarev is built on the identification of a local curve error reduction improvements to an input model based on simulation vs. real growth curves. Computational model simulation is very costly in terms of computer time. A prior scoring step was therefore included to predict local changes to the model likely to be interesting. Model refinement was restricted to selecting the edges to be removed from the cell-signaling submodels, although the addition of edges is also possible with the algorithm. The main steps in the model refinement algorithm are shown in Fig. 4*B*. (*SI Appendix 9* and *10*).

**Fig. 6.**
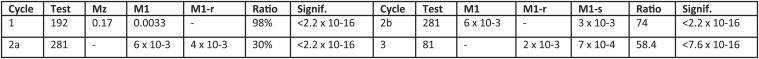
Experimental comparison of models. The number of test strains is the number of automated experiments used. M1-s, M-smart; M1-r, M1-random; Ratio, the relative reduction of error; Signif., the result of a pairwise Wilcoxon test of improved model over previous model (or M1-smart over M1-random for the last cycle).

In total, three closed-loop system biology cycles were executed. In the first cycle, the model Mz was semiautomatically improved using bioinformatic data to form M1. To assess the utility of cycle 1, we compared Mz and M1’s predictions with the empirical growth curves observed by Eve using a set of yeast gene deletant strains not used to form M1 (*SI Appendix 9*). The 192 strains selected for the experiments were taken from genes identified by CoRegNet as potentially involved in the diauxic shift (10) and randomly selected regulatory genes (kinases and transcription factors) (*SI Appendix 12*). The experimental results demonstrate that M1 is significantly better than Mz (Fig. 6).

The second and third closed-loop cycles differed from the first in including new planned experiments (Fig. 1*A*). In the second closed loop, inference tools were run to generate experiments, the experiments were executed, and the models were refined. Two sets of experiments were generated to improve M1: set (a) of 80 hypothesis-led experiments designed with our tools (AdactiveFB and CoRegMine), and set (b), consisting of 80 randomly selected experiments. Eve executed both sets of experiments. Model M1-smart was refined from M1 based on the results of hypothesis-led experiments, and model M1-random was refined from M1 based from the random experiments (Fig. 1 *A* and *B*). The motivation for generating two separate sets of experiments was to test the belief that hypothesis-led experiments (experiments designed to improve/test models) are more efficient in systems biology model development than random/high-throughput experiments (16). The M1-smart model has 298 nodes and 1,760 edges (Fig. 1*B*). We compared M1-smart and M1 using their predictions for 281 test strains. The results show that M1-smart is significantly more accurate at prediction than M1 (Fig. 6). We ensured the maximal improvement of M1-random and made the comparison between M1-random and M1-smart as rigorous as possible by selecting the 80 randomly selected genes from known yeast regulators (kinases and transcription factors) (*SI Appendix 13*). The M1-random model has 298 nodes and 1,778 edges (Fig. 1*B*). To compare M1 and M1-random, we applied their predictions on the same 281 test strains. M1-random was significantly better at prediction than M1 (Fig. 6).

In the third cycle, the M1-smart and M1-random models were compared by generation and execution of experiments. To generate the “crucial” experiments used to compare M1-smart and M1-random we applied the tool Adana to select 81 deletant strains with the largest predicted postshift growth rate disagreement between M1-smart and M1-random. We found that M1-smart was significantly better than M1-random (Fig. 6). We therefore concluded, as expected, that hypothesis-led experiments are more efficient at improving systems biology models than high-throughput/random experiments.

**Fig. 7.**
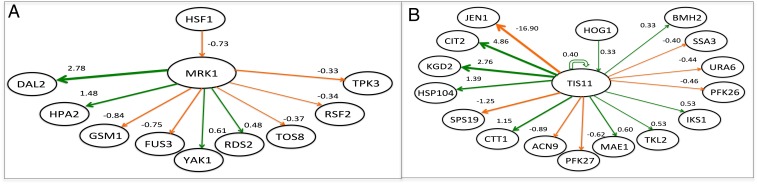
(*A*) Model fragment showing the connectivity of MRK1 and TIS11 in M1-smart. Nodes are shown with strengths >0.3. MRK1 is involved in modulating the diauxic shift and it mainly interacts with other kinases (FUS3, YAK1, and TPK3) and transcription factors (RDS2, TOS8, and RSF2) rather than enzymes—DAL2 an allantoicase is an exception. HSF1 is its sole parent; it is a trimeric heat shock transcription factor that has previously been implicated in the diauxic shift. (*B*) Model fragment showing the connectivity of TIS11 in M1-smart. TIS11 is mainly involved in directly controlling metabolic enzymes (CIT2, KGD2, SPS19, CTT1, PFK27, MAE1, TKL2, PFK26), especially those involved in sugar metabolism and the mitochondria. HOG1 is the sole parent of TIS11, it is a mitogen-activated protein kinase involved in osmoregulation. The strongest link is the repression of JEN1, a monocarboxylate/proton symporter of the plasma membrane that has previously been implicated in the diauxic shift.

An essential part of systems biology is the analysis of new models to provide biological insight (1). Our most accurate model, M1-smart adds a substantial amount of knowledge about the yeast diauxic shift: 92 extra genes (+45%) and 1,048 interactions (+147%). We used the Adana tool to rank the genes in terms of relative importance in the M1-smart model. To evaluate the biological insight possible from these additions, and to illustrate the biological utility of the knowledge generated by the system, we selected two genes highly ranked in M1-smart, but absent from Mz: MRK1 and TIS11 (*SI Appendix 14*). MRK1 (YDL079C) is homologous to human protein kinase glycogen synthase kinase-3 (GSK-3). Fig. 7*A* shows the fragment of M1-smart incorporating MRK1. GSK-3 genes are highly conserved and ubiquitous in eukaryotes and involved in differentiation, cell fate determination, and spatial patterning (17). These two highly homologous isoforms have been implicated in type II diabetes (Diabetes mellitus type 2), Alzheimer’s disease, inflammation, cancer, and bipolar disorder (17).

TIS11 is a member of the 12-O-tetradecanoylphorbol-13-acetate inducible sequence 11 family. TIS11 genes are involved in posttranscriptional gene regulation by micro-RNA (miRNA) and short interfering RNA (siRNA) (18, 19). Note that RNA processing is not explicitly included in M1-smart, and TIS11 was automatically incorporated as a putative transcription factor based on its zinc finger motif. This illustrates a strength of automating systems biology modeling: a human biologist would have excluded TIS11, yet its inclusion proved interesting, highlighting a possibly important role for RNA processing in the diauxic shift. Fig. 7*B* shows the fragment of M1-smart incorporating TIS11. In humans, changes in *TIS11* expression have been associated with both the suppression and promotion of cancer, and with autoimmune diseases (18).

Formal languages promote the reproducibility and reusability of results, and the exchange of information between human scientists and computer systems. We developed a suite of complementary ontologies to support the application of systems biology tools and their integration: (1) AdaLab-meta, an ontology for the description of metadata about datasets; (2) AdaLab, a domain ontology to represent relevant biological entities in systems biology; and (3) Eve-CV, a controlled vocabulary that defines typical Eve experiments and experimental conditions (*SI Appendix 15*). When combined these ontologies consist of ∼20,000 RDF (Resource Description Framework) triples. We collected and formalized in RDF all of the bioinformatic data used for this study to form a knowledge base of 1,301,017 RDF triples grouped in five separate RDF graphs: imported genes, genes annotations, genes expressions, Eve strains, and relevant metadata. The data are accessible via the linked data web interface (*SI Appendix 15*). We developed a dedicated communication mechanism SciCom (Scientific Communication) to communicate information about experiments to Eve. The requests for experiments and experimental results are stored in an RDF triple store in Manchester that consists of 10,187,417 RDF triples combined in two graphs.

## Discussion

The fundamental motivation for studying the diauxic shift in yeast (*S. cerevisiae*) is that it serves as a model for transformation in human cellular systems. It is therefore important to consider how well the methods can be scaled up for use in mammalian systems. This scaling up entails two main challenges: ensuring the same experimental reproducibility as is achievable in yeast, and scaling up the computational methods. We consider experimental reproducibility to be the most difficult of these challenges (20). For the scaling up of computational methods, the different parts of the software pipeline have different sensitivities to an increase in input network size (*SI Appendix 16*), but all of the methods scale polynomially, implying that the increase in size and complexity associated with the move to mammalian systems should be tractable with our approach.

We have successfully combined multiple systems biology software tools and laboratory robotics to execute three cycles of improvement for a model of the yeast diauxic shift. The cycles were not fully automated, as in the Robot Scientists Adam (12) and Eve (15), as the automation of systems biology is very much more complicated. However, full automation will be necessary to execute the hundreds or thousands of model improvement cycles required. The achievement of this full automation will require the software tools to be more robust and more modularly designed. Many of the software tools we have used are based on techniques originating in artificial intelligence (AI), especially machine learning: CoRegNet, CoRegMine, ELSA, ActiveFB, MinerLC*, Adarev, and Adana (Fig. 2). However, more advanced ideas from AI will be required to improve performance (21). For example, the tools have no high-level understanding of what they are doing, just as chess programs do not known that they are playing chess. One approach to providing them with such an understanding would be to give the system high-level goals to achieve, along with a higher-level planning ability. Another fundamental enhancement would be to give the AI tools the ability to communicate goals and intentions to human scientists.

In conclusion, we foresee a future in which combinations of software tools, laboratory automation, and human scientists will work together to create systems biology models that fully reflect and predict the underlying biology.

## Supplementary Material

Supplementary File
