# Supplementary material for: Closed-loop cycles of experiment design, execution, and learning accelerate systems biology model development in yeast
Source: Proc Natl Acad Sci U S A. 2019 Aug 16;116(36):18142–7. doi: 10.1073/pnas.1900548116 (PMC6731661; doi:10.1073/pnas.1900548116)
Supplement: Supplementary File [file pnas.1900548116.sapp.pdf]

Supporting information

Further information 1

1. Genomic scale metabolic model 3

References 3

2. Regulatory modelization 4

References 4

3. Full model simulation 5

References 6

4. Inference of the most influential Transcription Factors and Kinases for M1 7

References 7

5. Mz to M1 model extension 9

5.1. Initial genes importance ranking using CoRegNet 9

5.2. Ensemble learning of spanning arborescences 9

5.3. Refining M1raw into M1 by leave one out strategy 9

References 10

6. Backward vs. Forward experiments selection 11

F.1. Method 11

References 13

7. Graph mining for experiments selection 14

7.1. Co-regulation network and influence profiles 14

7.2. Discretization of influence profiles and node labeling 14

7.3. Identification of co-regulators genes candidates 15

References 17

8. Yeast culture and experimental methods 19

9. Model revision 20

9.1. Preparing the focus nodes ranking 20

9.2. Refinement of a focus gene local model 20

10. Instantiation for M1 revision 21

11. The Evaluation of model performance by post shift growth rate error 22

12. Mz vs. M1 evaluation strains 23

13. Random experiment strains 24

14. Model Analysis 25
References 25
15. Formalizing AdaLab Knowledge 26
15.1. AdaLab ontologies 26
15.2. SciCom 28
References 29
16. Scalability 30
References 31

1. Genomic scale metabolic model

The metabolic component of the models is a genome-wide metabolic model consisting of a set of species (metabolites) and reaction. The reactions between species are encoded using a stoichiometric matrix. Additionally, some reactions have an associated enzyme that performs the catalytic function of the reaction. Given that these enzymes may be operating as part of a complex, or be one of the many enzymes regulating the reaction, a set of rules, known as gene-protein-rules(GPR) encode this information. Given that we wish to simulate and derive observed biological quantities from these models, a set of constrains over the amount of mass allowed for each reaction is usually part of these model. Depending on the reaction directionality these constraints can go from (-inf, inf). If we set some of these constraints according to the experimental conditions (glucose consumption rate for example), and set biological objectives to maximize using a linear program, we obtain what is known as a Flux Balance Analysis solution. This solution contains the amount of mass for each reaction according to the pre-set constraints, and is an approximation to the metabolic state of the cell at steady- state. An extension of FBA, dynamic FBA, couples the solution of the FBA problem with a set of ordinary differential equations for metabolites we wish to allow to change concentrations in time.

The metabolic model selected was iMM904 (Mo, et al. 2009). This model has 8 compartments, 904 genes, 1228 metabolites, and 1412 reactions of which 395 are transport reactions.

References

Mo, M. L., Palsson, B., & Herrgård, M. J. (2009). Connecting extracellular metabolomic measurements to intracellular flux states in yeast. *BMC systems biology, 3*(1), 1.

2. Regulatory modelization

For a given set of regulatory genes **R** and a set of metabolic genes **M**, a regulatory model over all these genes $\mathbf{G} = \mathbf{R} \cup \mathbf{M}$ is described by a dynamic Bayesian network (DBN; Murphy, 2002) with 2 slices of time $M = (S, \Theta)$. The DBN structure $S = (\mathbf{R_t} \cup \mathbf{G_{\{t+1\}}}, \mathbf{E})$ is a directed acyclic graph over a nodes space composed of both regulatory genes at time t, and all genes in **G** at time $t + 1$. An edge $(a, b) \in \mathbf{E}$ describes a probabilistic dependency between the regulator gene $a \in \boldsymbol{R_t}$ at time t and a regulatory or metabolic target $b \in \boldsymbol{G_{\{t+1\}}}$ at time $t + 1$.

Each node in the model $x_i \in \mathbf{G_{\{t+1\}}}$ is associated to a parameter $\theta_i \in \Theta$ quantifying the probability of the node given its parents in the structure $P(x_i \mid parents(x_i) : S)$. The models are in a continuous space, with gene taking values in $[0; 1]$, and conditional linear Gaussian distributions (Lauritzen & Wermuth, 1984) are used to quantify these dependencies. Each θ_i is thus a pair $(\boldsymbol{\beta_i}, \sigma_i)$ with $\boldsymbol{\beta_i}$ a vector of $|parents(x_i)| + 1$ linear regression coefficients (one value per parent and an additional intercept), and σ_i the standard deviation describing the dependency uncertainty. Given parent values $\boldsymbol{p_i} \in [0; 1]^{\{|parents(x_i)|\}}$, a value $v_i \in [0; 1]$ of x_i follows a Gaussian distribution $N(\mu_i, \sigma_i)$, where we have $\mu_i = [\, \boldsymbol{p_i} \;\; 1 \,] \cdot \boldsymbol{\beta_i}$.

Fitting such a regulatory model, given a structure, was done through maximum likelihood learning over a normalized and dynamic $(t, t + 1)$ version of the Brauer dataset of micro-array data (Brauer et al., 2008). The dynamic transformation of the originally sized $t \times f$ time series is obtained by concatenating each consecutive set of row pair, using a sliding window of size 2, thus leading to a $(t - 1) \times 2f$ dataset size.

References

Murphy, K.P. (2002) Dynamic Bayesian networks: representation, inference and learning. PhD thesis, Computer Science, University of California, Berkeley, CA.

Brauer, M.J., Huttenhower,C., Airoldi,E.M., Rosenstein, R., Matese, J.C., Gresham, D., Troyanskaya, O.G., & David Botstein, D. (2008) Coordination of Growth Rate, Cell Cycle, Stress Response, and Metabolic Activity in Yeast. *Molecular Biology of the Cell* **19**, 352–367.

Lauritzen, S.L. and Wermuth N. (1984) Mixed interaction models. Technical Report R 84-8, Institute for Electronic Systems, Aalborg University.

3. Full model simulation

The regulatory part of the simulation, using only the regulatory model, is executed in a probabilistic way: the simulation takes the previous time step (or initial) vector of Gaussian distributions identified by their mean and standard deviations. The simulator then performs 1000 samples generation using a multivariate Gaussian regulatory model, and Gaussian inputs to finally compute distribution values at the next time step under the form of mean and standard deviations of the generated samples set.

The metabolic part of the simulation then takes the regulatory simulation metabolic gene mean values as input, together with a metabolic model (iMM904) and performs a dynamic flux balance analysis (DFBA) step using metabolic gene values as DFBA flux bound constraints, maximizing growth as the objective function. This step more precisely follows the CoRegFlux algorithm (Trébulle et al 2019, Banos et al., 2017):

- for each gene or group of genes of interest, look up for their corresponding GPR, which are in the form of AND and OR rules, select the expression levels of all the genes involved and use these levels in the following steps;
- for each "A AND B" rule, substitute it by a continuous relaxation min(A,B). Similarly, for each "A OR B" rule, substitute for max(A,B);
- we denote the result of the GPR rule evaluation for a gene of interest *I* as GPR_i
- the results of the continuous rule evaluation are then transformed using the function $\mathrm{softplus}(\mathrm{GPR_i}) = \ln(1 + \exp\{\theta + \mathrm{GPR_i}\}$. This transformation has a domain of $(0; \infty)$. It is a continuous non-linear function with a free parameter θ allowing the translation of a continuous value resulting from the evaluation of the GPR rules into flux constrains, as such big values will tend to infinity and by consequence un-constrain the flux through a given reaction. For reversible reactions, we set negative and positive flux constrains as $(-\mathrm{softplus}(\mathrm{GPR_i}), \mathrm{softplus}(\mathrm{GPR_i})$, for non-reversible reactions, we set to $(-softplus(GPR_i), 0)$ or $(0, softplus(GPR_i))$ correspondingly.
- with the readjusted flux bounds, for the time point *t* we now look into the concentration of the substrates of interest (Glucose and ethanol), and adjust the bounds for the corresponding uptake reactions in the metabolic model, that is, if v_c represents the current constraint bound (either from the model or from the result of the softplus function) and S_c represents the concentration of available substrate, we readjust the bound by $min(v_c, S_c)$;
- now that the fluxes constraints are adjusted for both genetic and metabolic limits, we solve the linear program represented by the FBA model with readjusted bounds. From this, we have outputs μ, the biomass yield, and the metabolites consumption/excretion rates v_o;
- we now update the biomass value X as $X_{t+1} = X_t \exp\{\mu\, \Delta t\}$, where $\mathrm{X_t}, \mathrm{X_{t+1}}$ represent biomass at times *t* and *t+1* respectively, and Δt is the difference between *t+1* and *t*. The metabolites concentration is updated by $S_{t+1} = S_t + \frac{v_o}{\mu\, X_t}(1 - \exp\{\mu\, \Delta t\})$, with$S_t$, S_{t+1} being the concentrations of the metabolites of interest at times *t* and *t+1*.

This step leads to the production of next time step biomass and metabolite levels (in the case of diauxic shift situation: less glucose if any, and more or less ethanol depending on the growth phase). Note that since both regulatory and metabolic simulators do not work on the same gene values space, an alignment is performed between regulatory and metabolic simulation, by projecting the regulatory [0; 1] values to a [x; y] metabolic values space thanks to a two parameters isomorphism (f, o) where f is a positive scale parameter and o is a negative offset parameter, so that we have: $x = o; y = f + o$.

After the metabolic simulation, the mean and standard deviations of each gene obtained at the regulatory step, together with metabolic simulation inferred metabolite levels are given to a metabolites regularization function. This function first computes for each gene in the model the number of activation rules which target it and for which clause body are true considering the current metabolite concentrations and other gene values, and the number of repression rules targeting it with true body. Then, if the number of activation (resp. repression) rules outnumbers the number of repression (resp. activation) rules, the gene is considered activated (resp. repressed) and thus its Gaussian distribution takes a mean of 0.99 (resp. 0.01) and a standard deviation of 0**.** If there is no dominance between activations and repressions, the gene state is not updated.

References

Trébulle P, Trejo-Banos D, Elati M (2019). *CoRegFlux: CoRegFlux*. R package version 0.99.23.

Banos, D.T., Trébulle, P., Elati, M. (2017) Integrating transcriptional activity in genome-scale models of metabolism. BMC Systems Biology 11 (Suppl 7):134 https://doi.org/10.1186/s12918-017-0507-0

4. Inference of the most influential Transcription Factors and Kinases for M1

Before the joint regulatory-metabolic model inference was conducted, a base set of influential Transcription Factors (TFs) and kinases was developed to guide the first round of experiments. For this step we used CoRegNet (Nicolle et al.,2015), which contains a set of tools for co-regulatory network inference and interrogation.

We followed the same protocol as in (Banos et al., 2017) for inferring general gene regulatory networks in Yeast:

- We selected a data set consisting of 247 experiments, 530 chips and 5520 probes. Gene expression is reported for each experimental condition by averaging out expressions of the chips corresponding to the same experimental condition (Faith et al.,2008).
- With this dataset we used CoRegNet(Nicolle et al., 2015) to infer a network of TF->Targets and (TF and Kinases)-> Targets. For TFs we considered all the genes marked as such in the Yeastract data base (Teixeira et al., 2006), we matched them to the genes present in the M3D data set for a total of 309 TFs. Kinases were identified through the yeast kinectome data base and matched to the genes present in M3D data set (258 kinases in total), expanding the list of potential regulators from 309 to 567.
- With the list of regulators and targets, and given the gene expression data set, CoRegNet uses a statistical algorithm to infer Regulator->Target interactions along with Regulator->Regulator interaction for co-regulation (Elati et al., 2007; Chebil et al., 2014).
- With the inferred networks (TF and TF+kinases), we computed the influence scores for two data sets. The influence score for a regulator is given by the adjusted difference of means:

$$I_i = \frac{\mu_A - \mu_R}{\sqrt{\left(\frac{s_a}{N_A} + \frac{s_R}{N_R}\right)}}$$

  where the influence of a regulator I_i is equal to the difference of the mean expression level of its activated targets μ_A and its repressed targets μ_R adjusted by their sample variances s_a ,s_R and the number of elements in each set N_A, N_R. This score can be recognized as the Welch's t-statistic, though the purpose of this score is not to assess statistical significance, but to represent the effect the regulators have over their activated and repressed targets.

We used the influence scores for the two networks calculated for two data sets. (Brauer et al, 2005) and (DeRisi et al, 1997). These scores were previously obtained and presented by (Banos et al.,2017), for the TF+kinases network. We also computed the influence scores for the TF only network. We ranked the regulators according to the absolute value of their influence scores for each sample, and used Robust rank aggregation (Kolde, et al., 2013) to obtain a consensus ranking of regulators. The top 40 TF from the TF only network, and the top 40 kinases of the TF+kinases network were selected for the first round of experiments in building M1.

References

Banos, D.T., Trébulle, P., Elati, M. (2017) Integrating transcriptional activity in genome-scale models of metabolism. BMC Systems Biology 11 (Suppl 7) :134 https://doi.org/10.1186/s12918-017-0507-0

Chebil, I., Nicolle, R., Santini, G., Rouveirol, C., & Elati, M. (2014). Hybrid method inference for the construction of cooperative regulatory network in human. *NanoBioscience, IEEE Transactions on, 13*(2), 97-103.

DeRisi, J. L., Iyer, V. R., & Brown, P. O. (1997). Exploring the metabolic and genetic control of gene expression on a genomic scale. *Science, 278*(5338), 680-686.

Kolde, R., Laur, S., Adler, P., Vilo, J. (2012) Robust rank aggregation for gene list integration and meta-analysis. Bioinformatics 15; 573-80.

Teixeira, M.C. et al., (2006) The YEASTRACT database: a tool for the analysis of transcription regulatory associations in *Saccharomyces cerevisiae*. *Nucleic Acids Research*, Volume 34, Issue suppl_1, 1 Pages D446–D451 https://doi.org/10.1093/nar/gkj013

5. Mz to M1 model extension

5.1. Initial genes importance ranking using CoRegNet

The Mz model contains far fewer genes, especially regulators, than predicted by bioinformatics. We therefore applied CoRegNet (Nicolle et al., 2014) to infer a regulatory network from a data set of 247 experiments for 5,520 probes. We looked for enriched associations in this network using as sources known regulatory interactions from the yeastract database (200,304 interactions; Texeira et al, 2006) and the *S. cerevisiae* Kinase and Phosphatase Interactome resource (262,354 possible P-P interactions), with a total of 567 potential regulators - TF and kinases. From these networks we computed the influence score ranking $\mathbf{R}^{567}_{\mathbf{coreg}}$ for the regulators, giving an order on the suspected impact of each of them on the diauxic shift process.

5.2. Ensemble learning of spanning arborescences

We first extended the regulatory Mz model in order to add genes that are predicted to be involved in the diauxic shift, but not yet present in this model. To this end we selected the top 40 transcription factors as well as the top 40 kinases in $\mathbf{R}^{567}_{\mathbf{coreg}}$, yielding a $\boldsymbol{G^{80}_{coreg}}$ gene set.

A raw extension of Mz, denoted M1raw, was produced by applying an ensemble network inference algorithm (Coutant & Rouveirol, 2017) to the (t, t+1) "dynamic" version of the Brauer dataset (in which each row is a concatenation of two consequent time series transcriptome data from the original Brauer dataset, leading to a (t, t+1) dataset), on the model space composed of the union of all Mz genes together with the $\boldsymbol{G^{80}_{coreg}}$ set, and using Mz model as a learning prior. From the dynamic version of the Brauer (Brauer et al, 2008) dataset, the first step of the algorithm is to compute a set of *components*, i.e. simple models that will be combined in the second part of the algorithm. Considering *m* components to learn, we first compute *m* local perturbations of the dynamic Brauer dataset by sampling (t, t+1) rows with replacement (a bootstrap aggregating or "bagging" strategy). Then, for each data resample D_u, a directed graph $G^u = (V, E^u)$, with V being the union of Mz nodes with $\boldsymbol{G^{80}_{coreg}}$ sets, is built by first randomly choosing $\frac{2}{|V|}$ undirected edges and then weighting them with the two directed scores:

- $s(a,b) = BGE(a_t \rightarrow b_{t+1}) + prior(a_t \rightarrow b_{t+1}) - BGE(\emptyset \rightarrow b_{t+1}) - prior(\emptyset \rightarrow b_{t+1})$
- $s(b,a) = BGE(b_t \rightarrow a_{t+1}) + prior(b_t \rightarrow a_{t+1}) - BGE(\emptyset \rightarrow a_{t+1}) - prior(\emptyset \rightarrow a_{t+1})$

, where BGE is the Bayesian Gaussian equivalent score (Geiger & Heckerman, 1994), for the evaluation of conditional linear Gaussian Bayesian network structures, and $prior$ is the prior score function privileging the Mz edges. Finally, each G^u is searched for its optimum spanning arborescence A^u using the Edmonds algorithm (Edmonds, 1968).

Once the *m* arborescence component models have been learned, the final step is to combine them into a *composite* model. This step is achieved by computing an expected model through considering the set of all possible edges in $V_t \times V_{t+1}$, where V_x denotes the set of genes V considered a time step x, and an expected edge score is computed by counting how often that edge was present in the A_u arborescences. A M1raw extension is thus defined by a set of unique edges $E \in V_t \times V_{t+1}$ having a non-zero occurrence value in the composite model computation, with the associated confidence score being proportional to occurrence in the arborescences.

5.3. Refining M1raw into M1 by leave one out strategy

The last step performed to produce the M1 model uses M1raw edges ranking (by decreasing occurrence score) to iteratively validate the edge impacts using a leave-one-out strategy on the dynamic version of the Brauer dataset.

More formally, starting from Mz model, and considering edges ranking by decreasing occurrence scores in M1raw denoted as $\vec{E}$, the iteration *i* of the refinement algorithm, considering the ith ranked edge $\vec{E}_i = (a_t \, , b_{t+1})$, executes the following:

- considering the extension of Mz obtained so far by previous iterations, named $\widehat{M1}$, a new candidate model $M1_{cand}$ is obtained by adding $\vec{E}_i$ to $\widehat{M1}$;
- for every of the 11 (t, t+1) individuals in the dynamic Brauer dataset, $\widehat{M1}$ and $M1_{cand}$ are both fitted with the whole dataset excluding this individual (a leave one out strategy), then prediction of b_{t+1} given genes at time t is performed with both models;
- the leave-one-out loop leads to 11 $\widehat{M1}$ vs. $M1_{cand}$ predictions of b_{t+1} given genes at time t. If more than 50% of these predictions (at least 6 over 11) are in favor of $M1_{cand}$, then update the current model $\widehat{M1} \leftarrow M1_{cand}$ for the next iteration. Otherwise, keep $\widehat{M1}$ and look at another edge in next iteration.

The final $\widehat{M1}$ model obtained after having iterated over $\vec{E}$ until the position of the last Mz edge corresponds to the $M1$ model.

References

Coutant, A. & and Rouveirol, C. (2017) Network Inference of Dynamic Models by the Combination of Spanning Arborescences. Journées Ouvertes en Biologie, Informatique et Mathématiques (JOBIM 2017), Lille, France.

Teixeira, M.C. et al., (2006) The YEASTRACT database: a tool for the analysis of transcription regulatory associations in *Saccharomyces cerevisiae*. *Nucleic Acids Research*, Volume 34, Issue suppl_1, 1 Pages D446–D451 https://doi.org/10.1093/nar/gkj013

Chebil, I., Nicolle, R., Santini, G., Rouveirol, C., & Elati, M. (2014). Hybrid method inference for the construction of cooperative regulatory network in human. *NanoBioscience, IEEE Transactions on, 13*(2), 97-103.

Brauer, M.J., Huttenhower,C., Airoldi,E.M., Rosenstein, R., Matese, J.C., Gresham, D., Troyanskaya, O.G., & David Botstein, D. (2008) Coordination of Growth Rate, Cell Cycle, Stress Response, and Metabolic Activity in Yeast. *Molecular Biology of the Cell* **19**, 352–367.

Geiger, D., & Heckerman, D. (1994). Learning gaussian networks. In *Proceedings of the Tenth international conference on Uncertainty in artificial intelligence* (pp. 235-243). Morgan Kaufmann Publishers Inc..

Edmonds, J. (1968). Optimum branchings. *Mathematics and the Decision Sciences, Part*, *1*(335-345), 26.

6. Backward vs. Forward experiments selection

Our active selection of experiments uses two strategies. The first one finds inconsistencies in model simulation by computing the mean deviation of regular (regulatory model to growth curves) *forward* simulations, and reversed growth curves to genes (*backward)* simulations, over the different EVE experimental conditions available. The second one identifies genes present in interesting co-regulation graph motifs for the diauxic shift, using graph abstraction. This section details the former strategy, the second one is detailed in section 7.

6.1. Method

A number of active strategies (Sverchkov & Craven, 2017) rank variables according to their discrepancy between observed and predicted states. Our revisions are focused on the regulatory part of yeast. However, we do not directly observe gene states in order to refine regulatory model and measure quality of its sub parts, but rather phenotypes of the form of growth curves. We therefore need to design a more sophisticated active strategy that adapts to the current experimental context of AdaLab and that heavily relies on both regulatory and metabolic simulations. The idea, given a KO gene, is to compare the forward simulated gene states time series to backward simulated time series that infer gene states consistent with observed growth curves.

6.2. Forward simulation

We call *forward simulation* the regular simulation performed from genes to phenotypes using both regulatory and metabolic simulators (section C of Further Information). This phase enables one to estimate a series of states for each gene in the DBN, as inferred in a regular way, as Gaussian distributions means and standard deviations.

The simulation starts from a first fully defined set of mean and standard deviation values for genes, using the Brauer dataset, metabolite initial concentrations (corresponding to the medium used in EVE experiments), initial optical density (OD) and OD to biomass factor. A forward simulation iteration is achieved by a sequence of 3 steps: 1) regulatory simulation, using DBN inference; 2) metabolic simulation, using DBN metabolic gene inferred distributions as metabolic simulator input, to get metabolite and biomass updates; 3) metabolite to gene regularization, using Mz network metabolite to gene rules. The knocked out gene given as parameter is assigned a value of 0 in every step of the simulation.

In order to exhibit model uncertainties, the forward simulations are compared to *backward* ones, namely reverse simulations using phenotypes evidence to infer genes state. Backward simulations heavily rely on metabolic gene series inference from phenotype curves to operate.

6.3. Metabolic gene series inference

One major step of the active selection workflow is to transform growth curve data from EVE into inferred metabolic gene series. This step is achieved by transforming: 1) optical density rates at each growth curve time point into flux bounds estimates at these points; 2) flux bounds into gene per reaction (GPR) bounds estimates at these points; 3) GPR bounds into metabolic gene bounds. The result of this step for a single growth curve is a set of metabolic gene time series, one for each gene.

Since the results of EVE experiments can be hierarchically organized, different levels of computations are possible for metabolic genes series inference:

- the well / experiment level;
- the set of wells with same plate and experimental conditions level;
- the set of experimental conditions level, involving all related plates.

Due to running time concerns, the choice was made to compute such inference on growth curves averaged over plates for each experimental condition. The average was computed by simply taking the mean of optical density points, after aligning times between plates. (A future more advanced solution could consider some (log)-normalization of curves before average, as is performed in some plots of EVE results folder, which would average the changes between time points.)

6.4. Backward Simulation

Backward simulation is executed using the results of metabolic gene inference from growth curves. The set of metabolic gene time series is first pre-processed to create evidence vectors for each time step. These evidences are built by scaling the values that are below 1000 (the infinite bound used in the cases where no information has been propagated to a metabolic gene during its inference) and clamping into the [0; 1] interval. In practice, this enables one to keep the original range for 70% of the gene values, and corresponds to some saturation level for the remaining 30%. The evidence preparation also includes an interpolation phase for the cases where the overall granularity misses some points. The final evidence vectors only contain the genes values that have an upper bound below 1000, and only the upper bounds are used as the means of the evidence distributions. The default standard deviation set for evidences is 0.1.

Due to the unavailability of inferred states for several genes, the function used for backward simulation is designed to deal with partial evidence, and thus the regulatory model is used for remaining genes inference. The result is a set of backward time series for all genes in the regulatory model, as Gaussian distributions means and standard deviations.

6.5. Forward vs backward simulation deviations

The main concept on which the active selection routine works is inconsistency between forward and backward simulations. Both simulations lead to the generation of an expected set of time series, one for each gene. The next step is to compute some deviations between the two outputs.

In our system, this step is achieved by computing the Kullback-Leibler divergence between forward and backward Gaussian distributions (using their means and standard deviations) for each gene and each time point. This leads to a divergence value for each (gene, time) pair.

F.6. Node revision / experiment requirement score

For a single EVE experiment result, a node revision score from a set of (gene, time, deviation) tuples is computed by first considering the difference of a gene deviation score and the average deviation score of its parents in the regulatory model for each time point, and then averaging this difference for each time point. The idea is to censure a node for its added uncertainty in the model, and not its absolute uncertainty which can be due to its parents.

For several EVE experiment results, each one leading to forward and backward simulations, as well as deviations computation for each (gene, time) pair, and the computation of above mentioned difference with parents, revision score is obtained per gene by averaging the (experiment, gene, time, deviation) quadruples over both experiment and time dimensions.

<u>The genes that are requested for knockout on Eve are the ones with maximum node revision scores from the above processes.</u>

6.7. Active selection for smart node identification from M1

In order to produce the set of smart nodes for the publication, the above processes were instantiated in the following way:

- first EVE experiments were requested using CoRegNet to rank top transcription factors and kinases predicted to involved in the diauxic shift process. This has led to the production of 137 single knockout strain experiment results;
- metabolic gene curves were inferred by backward inference from growth curve using reversed DFBA and flux bounds propagation;
- forward and backward simulations over the whole network were performed for the 137 strains, using the M1 model. The (f, o) alignment parameters between regulatory and metabolic simulators have been set to $(-1, 6)$ after having explored a grid of parameters on repeatedly simulating the Wild Type strain with the M1 model;
- per (experiment, gene, time) the delta score between forward and backward simulations was computed;
- per gene node revision scores were computed by aggregating over experiment and time
- the genes ranked by node revision score were merged with the graph mining nodes one (cf. Section G) to obtain the final set of top nodes, to form the smart experiments sent to Eve.

References

Sverchkov, Y., & Craven, M. (2017). A review of active learning approaches to experimental design for uncovering biological networks. PLoS computational biology, 13(6), e1005466.

7. Graph mining for experiments selection

7.1. Co-regulation network and influence profiles

We began by selecting a data set containing enough gene expression samples to obtain a representative network of gene regulation in yeast: Many Microbe Microarray Database (M3D) (Faith et al 2008). This database contains data from 247 experiments measuring gene expression under different conditions in microarray assays. The data were collated, normalized and averaged (in the case of replicates) for 5520 probes mapping onto ORF.

We used CoRegNet (Nicolle et al 2015) to infer a representative regulatory network for yeast. This network provides insight into the regulators that work together in the performance of a particular biological function. We enriched the network by searching the Yeastract database (Teixeira et al 2006) for known TF-target interactions, and the Biogrid database (Stark et al 2006) for known protein-protein interactions. The inferred CoRegNet network has a data structure extending beyond information about regulator-target and regulator-regulator cooperativity.

The produced bipartite graph allows the generation of a low-dimensional representation of the transcriptomic data (Nicolle et al 2012) using the regulator *influence* score.

The influence score accounts for the effect of a regulator on its targets according to the regulator-target relationships and additional data-sources integrated in the network as evidences. This measure is based on a Welch' t-test between the expression of the activated and repressed target genes in a given sample.

Vertices of the obtained co-regulation graph are co-regulators, and edges link two significantly cooperative co-regulators that are together necessary for the regulation of their target genes. To extract these co-regulator sets, all pairs of regulators which were found by h-Licorn (Chebil et al 2014) to be co-activators or co-inhibitors of at least one gene, in at least one GRN, are considered as potential co-regulators in the studied context. Then, only pairs that have a significant overlap of target genes using Fisher's exact test are predicted as co-regulators (with a 1% FDR control).

The influence profiles are computed for the co-regulators along the Diauxic shift course and are made of 12 timestamps (these data are derived from the (Brauer et al, 2008) Diauxic shift experiments). Each influence value represents the regulation activity of the considered co-regulator. The influence is high whenever the level of expression of targeted genes is consistent with the role (activator or inhibitor) and level of expression of the considered co-regulator.

The initial dataset was made of a co-regulation graph made of 180 co-regulators and a set of 156 co-regulators influence profiles. We reduced the co-regulators set to the co-regulators of the graph for which we had an influence profile. The resulting graph G(V, E) is made of 151 vertices and 1849 edges.

7.2. Discretization of influence profiles and node labeling

The influence profiles are used to label the vertices of G. In order to determine the set of labels for a given vertex, we first discretize the influence profile both along the time axis (Step 1) and the influence level (Step 2). We then generate labels from this discrete representation, as a set of binary variables representing the intervals the influence profile belongs to (Step 3).

Step 1: Each influence profile is split into 3 time windows ***W={B***, ***S***, ***A}*** corresponding to time laps occurring ***B***efore, during (***S***) and ***A***fter the Diauxic Shift. As shown in **Figure 1**, the time window ***B*** collects the influence values from time steps 1 to 7, ***S*** collects them from time steps 8 to 11 and ***A*** represents the last influence measure at time step 12.

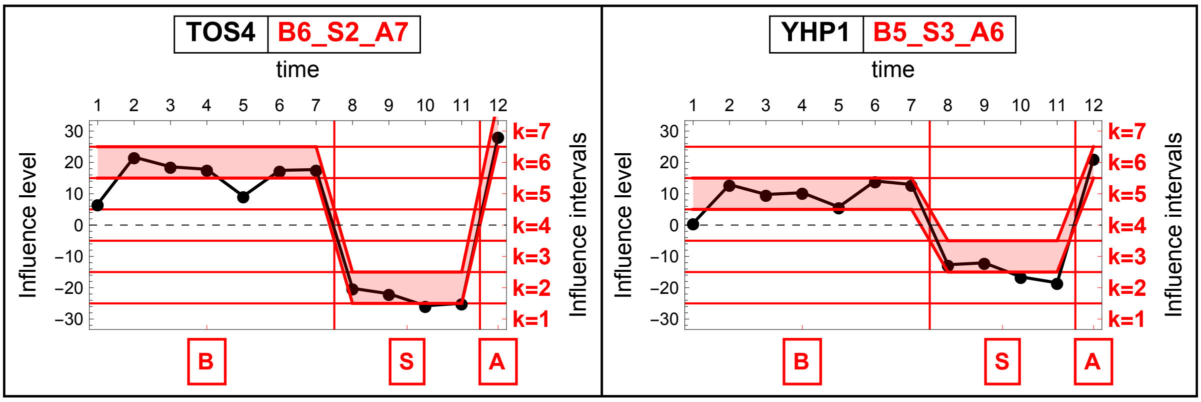

Figure 1 - Influence profiles description: Representation of the description of the discretized influence profiles of the co-regulators **TOS4** and **YHP1**. Black curves represent the original influence intensities along the time course. Segmentation bounds along the time axis define the 3 time windows $\boldsymbol{W}$, represented by vertical red lines. The discretization intervals of influence levels are represented in plain red and their identifying bin number reported on the right vertical axis. The binned description of the profile is represented in red (right part of the title)

Step 2: The influence levels are binned into 7 bins $\boldsymbol{k_w} \in \{1, \dots, 7\}$ (represented on the right axis in Figure 1). These bins have a closed range except the two extremal ones which represent open intervals. For each time window $\boldsymbol{W}$, the average influence level is used to determine $\boldsymbol{k_w}$. An influence profile is then represented by a sequence of 3 descriptive bins $(\boldsymbol{k_B}, \boldsymbol{k_S}, \boldsymbol{k_A})$.

<u>*Example:*</u> the influence profile of the YHP1 co-regulator (**Figure 1** - right) is represented by the triple $(\boldsymbol{k_B}, \boldsymbol{k_S}, \boldsymbol{k_A})$ = (5, 3, 6) where $\boldsymbol{k_B} = 5$ denotes that the average of influence profile values during the time window $\boldsymbol{B}$ belongs to the 5th bin. For the sake of clarity, the triple (5, 3, 6) is denoted as **B5_S3_A6**.

Step 3: Finally, each bin $\boldsymbol{k_w}$ is encoded as the set of all the 6 possible intervals of the form $\boldsymbol{W}1..\boldsymbol{k_{sup}}$ and $\boldsymbol{Wk_{inf}..7}$ with $7 > \boldsymbol{k_{sup}} \geq \boldsymbol{k_w}$ and $\boldsymbol{k_w} \geq \boldsymbol{k_{inf}} > \boldsymbol{1}$. A given interval is labeled by the ordered enumeration of its elements.

<u>*Example (labeling of a single interval):*</u> the $\boldsymbol{B2..7}$, *is* labeled by B234567.

<u>*Example (complete labeling of a vertex):*</u> at the time window **B**, the average influence level of the co-regulator YHP1 falls in the bin $\boldsymbol{k_B}$=5 (**B5**). The vertex YHP1 is then labeled with all the 6 intervals in which falls its discretized influence level, i.e. **B**234567, **B**34567, **B**4567, **B**567, **B**12345, **B**123456.

7.3. Identification of co-regulators genes candidates

The graph G'(V,E,L) labeled with the *labeling intervals* described above is submitted to the graph abstraction process in order to identify set of genes exhibiting similar patterns of influence profiles (i.e. in terms of labels) (Soldano et al. 2017). The graph abstraction enumerates (abstract pattern, abstract extension) couples where an abstract pattern is a subset of labeling intervals and an abstract extensions is a subset of vertices that all share the abstract pattern.

For the sake of clarity, we use in the following a condensed representation of the abstract pattern representing only the intersection of its vertices labeling intervals.

Example: in the example shown in Table 1, a vertex set (i.e. collection of co-regulators) shares a specific set of labeling intervals, For the time window **B**, the condensed abstract pattern is given by the intersection of the 6 labeling intervals resulting in the interval **B3**. The condensed representation of the complete abstract pattern obtained for all time windows is in this case **B3_S56_A2**.

Labels	B	1	2	**3**					S	1	2	3	4	**5**	**6**		A	1	**2**					
	B	1	2	**3**	4					S	2	3	4	**5**	**6**	7	A	1	**2**	3				
	B	1	2	**3**	4	5					S	3	4	**5**	**6**	7	A	1	**2**	3	4			
	B	1	2	**3**	4	5	6					S	4	**5**	**6**	7	A	1	**2**	3	4	5		
		B	2	**3**	4	5	6	7					S	**5**	**6**	7	A	1	**2**	3	4	5	6	
			B	**3**	4	5	6	7										A	**2**	3	4	5	6	7
Condensed			**B**	**3**									**S**	**5**	**6**			**A**	**2**					

Table 1: Condensed representation of an abstract pattern. Top: the set of labels (*i.e.* labeling union intervals) of a given abstract vertex set. Bottom: the condensed representation computed as the intersection of the abstract pattern intervals.

The abstract extension $\boldsymbol{X}$ of a pattern is defined as follows: it is the maximal vertex subset within the extension such that all vertices satisfiy some topological property in the subgraph $\boldsymbol{G_X}$ induced by **X**, called *core-property*. Here we use the 5-star core property which requires that the vertices of $\boldsymbol{G_X}$ have a degree of at least 5 or are connected to a vertex with a degree of at least 5. This 5-star core abstraction selects rather high degree vertices together with their neighbors.

Among the 160 abstract patterns found by the enumeration with MinerLC, we first looked for co-regulators sets with a steep transition during the shift, i.e. the ones with a high influence level before and low during the shift (named active-inactive profile - Ai for short), and those that exhibit an opposite influence profile: low before and high during the shift (named inactive-active profile - Ia for short). Allowed values for Ia (resp. Ai) influence profiles are B[1..4]_S[5..7]_A[1..4] (resp. B[5..7]_S[1..4]_A[5..7]). Influence profiles Ia and Ai are stated as *antagonists*.

Among all possible couples of (Ia, Ai) patterns, we identified the ones in which the union of the extensions of co-regulators is connected in the graph G. It results in a list of 144 couples deriving from 6 Ai and 24 Ia abstract patterns reported in **Table 2**.

Ai	B4_S4_A4<br>B45_S234_A456	B4_S34_A45<br>B456_S234_A4567	B45_S34_A456	**B45_S234_A45**
Ia	B3_S5_A3<br>B3_S6_A2<br>B3_S6_A12<br>B23_S6_A12<br>B23_S67_A12<br>B3_S56_A123	B3_S56_A3<br>B3_S5_A2<br>B3_S6_A123<br>B23_S6_A123<br>B23_S56_A12<br>B23_S56_A123	B23_S6_A1<br>B3_S6_A23<br>B23_S67_A1<br>**B3_S56_A2**<br>B23_S67_A123<br>B23_S567_A12	B2_S7_A1<br>B2_S67_A1<br>B3_S5_A23<br>B3_S56_A12<br>B3_S56_A23<br>B23_S567_A123

Table 2: Selected antagonist patterns which extensions are directly connected in the co-regulation graph. In bold: the pair of antagonist patterns selected to fix the list of candidate co-regulators for Eve experiments.

We first retained only couples of patterns that contain at least 3 co-regulators annotated to be involved in the Diauxic Shift in *Saccharomyces Cerevisiae*. We then selected the couple that both maximizes the *amplitude* and the *specificity* of the couple of patterns, as defined below.

Given the influence pattern $Bmin_B..max_B\_Smin_S..max_S\_Amin_A..max_A$ the amplitude $\delta(p)$ is defined as the maximal possible variation of the influence in the profile:

$$\delta(p) = \max(|min_B - max_S|, |max_B - min_S|) + \max(|min_S - max_A|, |max_S - min_A|)$$

The amplitude of a couple of pattern (p,p') is the sum of their individual amplitudes.

The specialization score sp(p) of each pattern p gives account of the sharpness of p. When the influence profile is sharp, the score is low and inversely. The score is computed as the sum of all the interval sizes:

$$sp(p) = \sum_{w=\{B,S,A\}} (max_W - min_W)$$

The specialization score of a couple (Ai, Ia) is the sum of their individual specialization scores.

Example:

In the patterns B45_S234_A45 and B3_S56_A2, the amplitudes are:

$$\delta(\text{B45\_S234\_A45}) = \max(|4-4|, |5-2|) + \max(|2-5|, |4-4|) = 6$$

$$\delta(\text{B3\_S56\_A2}) = \max(|3-6|, |5-2|) + \max(|2-5|, |6-2|) = 7$$

and the specialization scores are:

$$sp(\text{B45\_S234\_A45}) = (5-4) + (4-2) + (5-4) = 4$$

$$sp(\text{B3\_S56\_A2}) = (3-3) + (6-5) + (2-2) = 1$$

Given those scores, we then select the couple of patterns (Ai, Ia) that satisfies the best compromise in terms of specialization and amplitude of their patterns. We take the top-40 pairs of patterns with higher amplitudes and then select the couple of pattern having the lower specialization score: the pair (**B45_S234_A45, B3_S56_A2**) (shown in Figure 2c-bottom of the main article). The co-regulators genes linked in G for which one end is in the Ai pattern extension (co-regulators/vertex set) and the other in the antagonist Ia pattern extension are proposed as candidate genes for experiments using Eve.

References

Faith, Jeremiah J and Driscoll, Michael E and Fusaro, Vincent A and Cosgrove, Elissa J and Hayete, Boris and Juhn, Frank S and Schneider, Stephen J and Gardner, Timothy S (2008). Many Microbe Microarrays Database: uniformly normalized Affymetrix compendia with structured experimental metadata. *Nucl. Ac. Res.*, **36**, 866-870.

Nicolle, Rémy and Radvanyi, François and Elati, Mohamed (2015). Coregnet: reconstruction and integrated analysis of co-regulatory networks. *Bioinformatics*, **31**(18).

Teixeira, Miguel C and Monteiro, Pedro and Jain, Pooja and Tenreiro, Sandra and Fernandes, Alexandra R and Mira, Nuno P and Alenquer, Marta and Freitas, Ana T and Oliveira, Arlindo L and Sà-Correia, Isabel (2006). The YEASTRACT database: a tool for the analysis of transcription regulatory associations in Saccharomyces cerevisiae. *Nucl. Ac. Res*. **34**, 446-451.

Stark, Chris and Breitkreutz, Bobby-Joe and Reguly, Teresa and Boucher, Lorrie and Breitkreutz, Ashton and Tyers, Mike (2006). BioGRID: a general repository for interaction datasets. *Nucl. Ac. Res*. **34**, 535-539.

Nicolle, Rémy and Elati, Mohamed and Radvanyi, François (2012). Network transformation of gene expression for feature extraction. *Machine Learning and Applications (ICMLA) 2012 11th International Conference on*. **1**, 108-113.

Chebil, I and Nicolle, Rémy and Santini, G and Rouveirol, Céline and Elati, Mohamed (2014). Hybrid method inference for the construction of cooperative regulatory network in human. *IEEE transactions on nanobioscience*, **13**, 97-103.

Brauer, M.J., Huttenhower,C., Airoldi,E.M., Rosenstein, R., Matese, J.C., Gresham, D., Troyanskaya, O.G., & David Botstein, D. (2008) Coordination of Growth Rate, Cell Cycle, Stress Response, and Metabolic Activity in Yeast. *Molecular Biology of the Cell* **19**, 352–367.

H. Soldano, G. Santini, and D. Bouthinon. Formal concept analysis of attributed networks. In R. Missaoui, S. Obiedkov, and S. Kuznetsov, editors, Formal Concept Analysis in Social Network Analysis, Lecture Notes in Social Networks, pages 143–170. Springer, 2017.

8. Yeast culture and experimental methods

Haploid yeast strains from the EUROSCARF deletion collection or a wildtype BY4741 strain were cultured overnight in 250µL YPD medium (Sigma) at 30°C in a round bottom 96 well plate (Nunc). Plates were then centrifuged at 2000rpm for 1 minute, before Eve's Agilent Bravo and MultiDrop liquid dispensers were used to aspirate culture medium and wash the pellets in 250µL PBS (Sigma). Yeast cells were then centrifuged at 2000rpm for 1 minute before the Bravo and MultiDrop were used to remove PBS and the pellets were resuspended in 250µL of growth medium comprising YNB supplemented with L-methionine, uracil, L-leucine and L-histidine (625µg/ml, Sigma) and 0.25% dextrose (Fisher).

The Bravo was used to dilute these stock cultures to a starting optical density (OD) of ~0.07 in growth medium, and transfer 25uL cultures from each well of the 96 well plate to one quadrant of an uncoated 384 well plate (Corning). Quadrants on the outermost rows and columns of each plate were filled with growth medium that had not been inoculated – this avoided edge effects due to evaporation and served as a control for any contamination of growth medium.

To measure glucose consumption yeast was set up as described above for growth experiments, but with a varying layout. Up to 8 strains were added to a 384 well plate in columns containing eight quadrants (32 wells). Two further columns were added, containing a negative control (YNB medium without any added dextrose) and positive control (YNB medium with 0.25% dextrose).

Following plate setup, Overlord 2 was programed to facilitate transfer of the experimental plate between Eve's Liconics incubator (30°C), and a BMG Polarstar platereader for OD measurements at 560nM. The interval between readings was typically set to 15 minutes, with one of Eve's Mitsubishi robot arm used to remove and replace the lid of each plate prior to a reading, allowing oxygen in the air present in the plate to be replenished. Experiments had duration of up to 70 hours. Individual plates were barcoded, allowing their workflow to be tracked and permitting up to eight plates per experiment.

The glucose consumption experiments tracked growth, as described above, but also incorporated the addition of a 20µL of a resorafin-based glucose assay (A22189 – Thermo Fisher) to one row of wells using Eve's Bravo at up to 11 time points. This assay provided a colorimetric readout that could be detected by the same OD560 measurements used to track yeast growth. Glucose measurements for each strain were quantified using the positive and negative controls for each timepoint. At the end of the experiment, corresponding growth curves for each strain were calculated using the OD measurements in the remaining wells to which the assay had not been added.

9. Model revision

The algorithm described as "model revision" in the main paper aims at, starting from a model to update, find a local improvement of this model based on simulation vs. reality growth curve errors reduction. Since the simulation of a full yeast model involves communication between the regulatory and metabolic simulators takes significant time, a prior scoring step is added in order to determine which local candidates of a model are more likely to be interesting - based on knowledge base information and other measures easily computable in the model itself. Also, in order to evaluate the importance of selecting a set of genes over another on revision performances, the proposed algorithm is based on the concept of *focus* genes. This set of genes determines which set of local candidates are generated then potentially tested for error reduction and thus definitely applied to the model.

9.1. Preparing the focus nodes ranking

More formally, let $\boldsymbol{F}$ be a set of focus nodes "around" which revisions have to be considered from a regulatory model M. For each gene $g \in \boldsymbol{F}$, the strain obtained by knock-outing it is simulated using M to get a post shift growth rate error $error(M, g)$. In our system the error is obtained by computing the difference between the real post shift growth rate and the simulated post shift growth rate for the given strain. These errors are ranked by decreasing absolute values in order to form a permutation $\overrightarrow{\boldsymbol{F}}$ of $\boldsymbol{F}$.

The revision steps then consists in iteratively applying a local refinement algorithm to these nodes in $\overrightarrow{\boldsymbol{F}}$ order.

9.2. Refinement of a focus gene local model

9.2.1. Generating focus gene local candidates

Let $g \in \overrightarrow{\boldsymbol{F}}$ be the gene under focus for a new iteration, each previous gene in $\overrightarrow{\boldsymbol{F}}$ having been focused on, and denoting as $\widehat{M}$ the model obtained from M by keeping all validated revisions so far. The first step of the local refinement routine is to generate local perturbations of the model to revise, denoted as the $\widehat{M}_{\leftrightarrow}$ model set, using the sign of $error(M, g)$. More precisely, if the sign is such that real curve has higher post shift growth rate than the simulated one, the algorithm interprets this as a knockout having too much impact in $\widehat{M}$. Thus, the space of generated candidates $\widehat{M}_{\leftrightarrow}$ is made of: 1) models obtained by removing one child to g in $\widehat{M}$; 2) models obtained by adding one parent regulator to one child of g in $\widehat{M}$. If the sign is such that real curve has lower post shift growth rate than simulated one, the algorithm interprets this as a knockout having too little impact in $\widehat{M}$. Thus, the space of generated candidates $\widehat{M}_{\leftrightarrow}$ is made of: 1) models obtained by adding one child to g in $\widehat{M}$; 2) models obtained by removing one parent regulator to one child of g in $\widehat{M}$.

9.2.2. Local candidates prior evaluation

In order to limit the number of simulations to perform, which can be costly, and since the number of generated candidates can be important, a local candidates filtering phase is first conducted using non-simulation evaluation sources to estimate the relevance of adding or removing an edge from $\widehat{M}$.

More precisely, each local candidate $\widehat{M}_{\leftrightarrow}$ differs from $\widehat{M}$ by a single operation consisting in either adding or removing an edge. Each considered operation is thus independently evaluated using different scores including:

1. a measure involving the divergences of parent and child knockout errors with observed experimental results (whenever the corresponding experiment is available);

2. (for removal only) a measure privileging removal of non Mz edges;
3. a measure assessing the topological impact of removing or adding each edge on the parent to child new distance in the graph (measuring a redundancy or a scarcity in paths);
4. a measure involving CoRegNet regulators to non-regulator descendant relationship to produce a direct dependency score, by a product of f-measures on the quality of removing (resp. adding) as much possible false (resp. true) descendant to the parent of removed (resp. added) edge, and on the quality of the child descendant to have already adequate descendants.

The neighbor scores are used to produce a new models ranking $\overrightarrow{\widehat{M}_{\leftrightarrow}}$, from which the top k are used for simulation of growth curves.

9.2.3. Top local candidates simulation for error gain estimation

Let $\overrightarrow{\widehat{M}_{\leftrightarrow}[1;\mathrm{k}]}$ be the set of top k candidate models having the best prior score in previous step. Each of these candidates is then simulated for error gain comparatively to $\widehat{M}$. The simulation of a candidate impact $\overrightarrow{\widehat{M}_{\leftrightarrow}[\mathrm{u}]}$ with $1 \leq \mathrm{u} \leq \mathrm{k}$ is approximated by considering the simulation of every parent p of the corresponding operation edge, both in $\widehat{M}$ and $\overrightarrow{\widehat{M}_{\leftrightarrow}[\mathrm{u}]}$, and their respective error against real curves $error(\widehat{M}, p)$ and $error(\overrightarrow{\widehat{M}_{\leftrightarrow}[\mathrm{u}]}, p)$. Then, an error gain is computed for each parent p:

$$\delta_p^u = error\left(\overrightarrow{\widehat{M}_{\leftrightarrow}[\mathrm{u}]}, p\right) - error(\widehat{M}, p),$$

and a global error gain estimation $\Delta^u = \sum_p \delta_p^u$ is computed. The uth model candidate is finally assigned its score Δ^u if it is negative (thus denoting an error reduction) and if no focus node dealt with in previous algorithm iterations have a positive error gain (thus denoting an error increase). In the other case, it is discarded from possible model improvements.

The remaining model candidates are finally ranked according to their increasing Δ^u scores (better models have negative error gains).

9.2.4. Validation and application of local operations

This final ranking is used to apply revisions starting with the most promising ones, iteratively validating the proposed changes as long as new predictions in the updated models are better than previous ones - in terms of post shift growth rate error reduction gain.

10. Instantiation for M1 revision

The model revision phase described in the main paper follows the revision steps explained in this appendix, with the following specificities:

- M1 is used as the initial model for both smart and random revisions;
- Smart revision uses the actively selected set of genes as focus nodes set, while random revision uses the randomly selected set of genes;
- Since M1 adds many edges from the Mz model, the choice was made to restrict the revised

11. The Evaluation of model performance by post shift growth rate error

The comparison between two regulatory models M_a and M_b is based on post shift growth rate differences between each model simulation and the ones inferred from EVE growth curves, for a given set of strains situations described by a set of single knock-out genes $\boldsymbol{G}$.

The first step toward models comparison is to compute all simulated post shift growth rates errors with real growth curves, for all $g \in \boldsymbol{G}$, leading to the production of error sets $\{error(M_a, g)\}_{g \in \boldsymbol{G}}$ and $\{error(M_b, g)\}_{g \in \boldsymbol{G}}$, where $error(M_i, g)$ is obtained by subtracting the simulated post shift growth rate of M_i under the g single knock-out condition in the observed Eve experiment curve post shift growth rate for the same condition. A negative error value thus indicates an improvement since it exhibits an error reduction for this condition.

The overall or *cumulative absolute* model M_i error for a set of single knock-out genes $\boldsymbol{G}$ is obtained by summing all errors in the corresponding error set: $error(M_i, \boldsymbol{G}) = \sum_{g \in \boldsymbol{G}} error(M_i, g)$. We use the difference between two model overall errors to estimate the overall improvement or decline of performances from one model to another. Namely: $gain(M_a \rightarrow M_b, \boldsymbol{G}) = error(M_b, \boldsymbol{G}) - error(M_a, \boldsymbol{G})$. Again, a negative gain indicates an improvement since it is associated to an overall error reduction.

The overall error and improvement are insufficient to characterize the differences between two models. Therefore per-strain measures are also considered, through per-strain *gain*:

$$gain(M_a \rightarrow M_b, g) = error(M_b, g) - error(M_a, g).$$

This per-strain gains enables one to compute statistical significance tests by considering each strain as a sample and considering the per sample pairwise statistical test results to measure how much significant the dominance of one model over the other, given the number of time it has lower error. In practice, we use Welch t-test and Wilcoxon pairwise tests.

12. Mz vs. M1 evaluation strains

Mz and M1 were compared relatively to the first Eve experiment batches. These batches consist in single knock-outs of: 1) the set of top TFs and kinases as identified by CoRegNet; 2) the set of 80 random genes used for evaluation of smart vs. random revision contexts. Overall, 200 unique strain experiments are used:

YAL017W YAL040C YAL051W YBL009W YBL016W YBL054W YBR020W YBR059C YBR066C YBR093C YBR150C YBR240C YBR297W YCL024W YCR019W YCR079W YCR091W YDL020C YDL025C YDL036C YDL042C YDL075W YDL079C YDL159W YDL214C YDR009W YDR074W YDR096W YDR146C YDR169C YDR216W YDR226W YDR277C YDR423C YDR436W YDR448W YDR451C YDR477W YDR490C YDR507C YER028C YER095W YER123W YFL001W YFL044C YFR034C YGL021W YGL035C YGL096W YGL179C YGL180W YGL197W YGL208W YGL209W YGL215W YGR044C YGR063C YGR067C YGR097W YGR108W YGR109C YGR123C YGR161C YGR169C YHL032C YHR006W YIL036W YIL042C YIL045W YIL050W YIL101C YIL113W YIL122W YIR026C YJL057C YJL089W YJL098W YJL106W YJL141C YJL157C YJL164C YJL165C YJR059W YJR066W YJR144W YJR147W YKL001C YKL038W YKL166C YKR021W YLL019C YLR079W YLR113W YLR133W YLR136C YLR165C YLR174W YLR183C YLR260W YLR448W YML027W YML113W YMR014W YMR053C YMR104C YMR139W YMR182C YMR199W YMR280C YMR291W YNL098C YNL183C YNL289W YNL298W YNR012W YNR031C YOL016C YOR014W YOR101W YOR113W YOR290C YOR344C YOR363C YPL042C YPL089C YPL109C YPL129W YPL137C YPL179W YPL212C YPL230W YPL256C YPR106W YPR115W YPR119W YPR120C YPR193C YRP115W YAL054C YBR083W YBR125C YBR159W YCL040W YDL066W YDL070W YDL188C YDR034C YDR099W YDR191W YDR247W YDR310C YDR466W YDR520C YER068W YER088C YER109C YGL071W YGL254W YGR019W YGR192C YHR092C YHR143W YIL002C YIL038C YIR018W YJL045W YJL110C YJL128C YJL187C YJR127C YKL188C YKL198C YKL217W YKR009C YKR101W YLL026W YLR025W YLR284C YLR357W YLR418C YML081W YMR246W YNL097C YNL117W YNL154C YNL257C YNL309W YNL314W YNR001C YNR063W YOL051W YOL089C YOL147C YOR173W YOR213C YOR233W YPL112C YPL150W YPL177C YPR008W.

13. Random experiment strains

The set of random genes has been built in the following way. First, all yeast regulators (567 split into TFs and kinases), as well as Mz metabolic genes (152) have been gathered to produce a set of 719 interesting genes to knock-out. Then, a random permutation was produced by sampling (without replacement), and the first 80 genes with viable knock-outs were chosen to be part of the Eve experiments random strains batch. The detailed set of random experiment strains is composed of:

YAL054C YBR059C YBR066C YBR083W YBR125C YBR159W YBR240C YCL040W YDL025C YDL066W YDL070W YDL188C YDR034C YDR099W YDR191W YDR247W YDR310C YDR466W YDR520C YER068W YER088C YER109C YGL021W YGL071W YGL209W YGL254W YGR019W YGR063C YGR192C YHL032C YHR006W YHR092C YHR143W YIL002C YIL038C YIR018W YJL045W YJL110C YJL128C YJL187C YJR059W YJR127C YKL038W YKL188C YKL198C YKL217W YKR009C YKR101W YLL019C YLL026W YLR025W YLR284C YLR357W YLR418C YML027W YML081W YMR246W YNL097C YNL117W YNL154C YNL257C YNL309W YNL314W YNR001C YNR063W YOL051W YOL089C YOL147C YOR173W YOR213C YOR233W YPL089C YPL112C YPL150W YPL177C YPR008W YPR106W YPR115W.

14. Model Analysis

MRK1 (YDL079C) is homologous to human protein kinase glycogen synthase kinase-3 (GSK-3), and Drosophila Zeste-White3/Shaggy. It is one of a family of four ser/thr protein kinase homologs: MRK1, YGK3, RIM11, and MCK1. MRK1 and YGK3 arose through genome duplication, as did RIM11 and MCK1 (Kassir et al., 2006). AdaLab selected all four homologs for addition to M1-smart. However, MRK1, at position 9, is ranked by far the highest in importance: MCK1 – 215, RIM11 - 219, and YGK3 - 545. Rim11 and MCK1 have previously been identified as important genes: Rim11 is 'essential for entry into meiosis', and MCK1 is 'essential for growth at elevated and low temperatures' (Kassir et al., 2006). In contrast, MRK1 has been relatively little studied. GSK-3 genes are highly conserved and ubiquitous in eukaryotes. They have been implicated in differentiation, cell fate determination, and spatial patterning (Ali et al., 2001). In human there are two highly homologous isoforms: alpha (GSK3A), and beta (GSK3b). These have recently been the subject of considerable research, as they have been implicated in several important diseases: Type II diabetes (Diabetes mellitus type 2), Alzheimer's Disease, inflammation, cancer, and bipolar disorder (Ali et al., 2001). AdaLab's new understanding of the mechanistic role of MRK1 in the yeast diauxic shift should inform the development of drugs that target GSK3A and GSK3b.

The second gene selected for analysis was TIS11 (YLR136C), the 13th most important gene in M1-smart. TIS 11 is a member of the 12-O-tetradecanoylphorbol-13-acetate (TPA) inducible sequence 11 family. TIS11 genes are involved in posttranscriptional gene regulation by micro-RNA (miRNA) and short interfering RNA (siRNA) (Baou et al. 2009; Ma & Herschman, 1995). Note that RNA processing is not explicitly included in M1-smart, and TIS11 was automatically incorporated as a putative transcription factor based on its zinc finger motif. The inclusion of TIS11 illustrates the strengths and weaknesses of automating systems biology modelling. A human biologist would have excluded TIS11 on the reasonable grounds that it is neither a kinase nor a transcription factor. Nevertheless, the inclusion of TIS11 is very interesting, and points to RNA processing having an important role in the diauxic shift. Interestingly, TIS11 was originally manually classified as a transcription factor (Baou et al. 2009). In yeast TIS11 expression is repressed by glucose, and it has been reported that gene disruption causes an 'alteration in metabolism', but 'does not effect viability, growth in rich or synthetic medium' (https://www.yeastgenome.org/). TIS11 has a paralog, CTH1, which arose from genome duplication, but this was not incorporated in M1-smart. Human TIS11 was first isolated and cloned after stimulation TPA or insulin. The insulin link may be significant given yeast TIS11's role in the diauxic shift (Baou et al. 2009). In humans some of the main mRNA targets are TNF-α, IL-3, IL-2, IL-6, IL-10, and TIS11 itself (Baou et al. 2009) – self-stimulation is also found in M1-smart, see Fig. 4c. Changes of TIS11 expression levels have been associated with both suppressing and promoting cancer, as well as autoimmune diseases (Baou et al. 2009).

References

1. Baou, M., Jewell A., Murphy, J.J. (2009) TIS11 Family Proteins and Their Roles in Posttranscriptional Gene Regulation. Journal of Biomedicine and Biotechnology ID 634520, 11 pages.
2. Kassir Y, Rubin-Bejerano I, Mandel-Gutfreund Y. (2006) The Saccharomyces cerevisiae GSK-3 beta homologs. *Curr Drug Targets* 7(11):1455-65
3. Ma, Q., Herschman, H.R. (1995) The yeast homologue YTIS11, of the mammalian TIS11 gene family is a non-essential, glucose repressible gene. Oncogene, 10, 487-94.

15. Formalizing AdaLab Knowledge

Formal languages readable by both humans and machines are essential for the exchange of information between human scientists and AI systems. Formal languages promote semantic clarity, which in turn supports the free exchange of scientific knowledge, and simplifies scientific reasoning. The first step in formalizing knowledge is to define an explicit ontology, i.e. to describe what exists. We developed a suit of complementary ontologies to support the AI tools: 1) AdaLab-meta an ontology for the description of metadata about datasets, 2) AdaLab a domain ontology to represent relevant system biology biological entities, and 3) Eve-CV (Eve experiments control vocabulary) that defines typical Eve experiments and experimental conditions (Supp AdaLab ontologies). AdaLab ontologies enable the recording, storage and the exchange of AdaLab data between different components of the AdaLab framework, and also between human and robotic scientists. When combined these ontologies consist of ~20,000 RDF (Resource Description Framework) triples. We collected and formalized in RDF all the bioinformatic data used for this study. This combined knowledgebase of 1,301,017 RDF triples grouped in five separate RDF graphs: genes imported, genes annotation, genes expression, Eve strains and knowledge base descriptions (metadata). Access to the data is available via the linked data web interface: http://rdf.adalab-project.org/resource/[domian name], where each domain has unique URI pattern. All domains are described in ADALAB-META ontology (Supp AdaLab ontologies). To communicate information about experiments from Paris and Lille to Manchester, we developed a dedicated communication mechanism SciCom (Scientific Communication, see Supp SciCom). The requests for experiments and experimental results are stored in an RDF triple store in Manchester that consists of 10,187,417 RDF triples combined in two graphs. For example, access to the experiment 1 is available at http://rdf.adalab-project/resource/experiment/1.

15.1. AdaLab ontologies

We developed a suit of complementary ontologies to support the AdaLab framework:

1) the AdaLab-meta ontology for the description of metadata about datasets,
2) the AdaLab domain ontology to represent relevant system biology biological entities,
3) Eve-CV (Eve experiments control vocabulary) that defines typical Eve experiments and experimental conditions.

We followed best practices in ontology development and OBO (Open Bio Ontologies) Foundry recommendations[1]. We used the standard Web Ontology Language OWL as the most suitable for the purposes of the project among the recommended[2]. We also used another recommended standard language - the Resource Description Framework (RDF)[3]. Any data record is encoded as a collection of the subject-predicate-object triples.

AdaLab ontologies enable the recording, storage and the exchange of AdaLab data between different components of the AdaLab framework, and also between human and robotic scientists. When combined these ontologies consist of ~20,000 RDF (Resource Description Framework) triples. We also collected and formalized in RDF all the bioinformatic data used. This combined produced a knowledgebase of 1,301,017 RDF triples.

---

[1] http://www.obofoundry.org/
[2] https://www.w3.org/OWL/
[3] https://www.w3.org/RDF/

These ontologies have value as stand-alone products, and they also can be seamlessly imported within one AdaLab-Core ontology.

15.1.1. AdaLab-meta ontology

The AdaLab framework relies on the usage of many datasets by the machine learning and biomodeling components, and also outputs new datasets. Therefore, it is important to describe these resources in terms of their formats, features, provenance, and quality. An efficient way to do it is through a defined set of metadata.

We reviewed several meta-data resources, but none of them fully satisfied the requirements of the project. We therefore had to design our own metadata set by incorporating terms from other metadata sets. We re-used several relevant resources for AdaLab meta-ontology:

- The Data Mining Optimisation Ontology (DMOP) describes such relevant entities as *Dataset*, *Feature* and *DataTable* [1]. DMOP ontology was designed to support the selection of algorithms, models, and workflows in a view of optimizing the data mining process and the quality of the mined hypotheses (models or pattern sets) [2].
- DC (Dublin Core) defines such entities as *Creator, Licence, Format.* We mapped relevant DC terms to AdaLab-meta specific terms. For example *DC: Creator* is mapped to *Adalab-meta: ResourceCreator.*
- Prov-o[4] ontology covers data provenance and processing terms.

Metadata terms defined in our meta-ontology include such terms as *dataset title, abstract, (associated) publication, creator,* and *quality*. The terms are explicitly linked to the resources they were imported from. AdaLab-meta ontology was used for the description of datasets in a human readable form and also in machine processable index cards.

Availability: https://bioportal.bioontology.org/ontologies/ADALAB-META

15.1.2. AdaLab domain ontology

The main aim of the AdaLab domain ontology (or simply AdaLab ontology) is to integrate the ontology(-ies) required for the management of various data sources with standardized ontology describing the biological interaction networks. After discussions with our project's partners regarding the project requirements for knowledge representation a decision was made to re-use popular biological BioPAX-based models [2]. AdaLab data model is aligned with Integration Network Ontology (INO) which is a restructured BioPAX ontology following OBO standards and expressed in OWL 2 language [1,3]. We used INO as an upper-level ontology of AdaLab domain ontology and extended it by our project-specific terms.

The three most populated subtrees in the class hierarchy of the AdaLab ontology are rooted to the upper-level classes: *information content entity, material entity* and *process*. The *information content entity* class links the ontology with the 'raw' datasets described by the AdaLab-meta ontology. The *material entity* class has subclasses representing different biological concepts such as: *organism, gene, genome*. The key subclass of the class *process* is the *interaction* process.

Availability: https://bioportal.bioontology.org/ontologies/ADALAB

---

[4] https://www.w3.org/TR/prov-o/

15.1.3. Eve-CV

A controlled vocabulary for the exchange of information about Eve experiments (Eve-CV) enables efficient communication between various components of the AdaLab system, e.g. between automated laboratory Eve (Robot Scientist) and machine learning (ML) for experiment selection.

Eve-CV defines the most essential entities pertinent to experiments run on Eve. Each experiment has unique ID, and the key information associated with each experiment is a set of *experimental factors* and a set of *experimental outputs*.

The class experimental factor has two subclasses:

- *well factor*, an experimental factor pertinent to a specific well, e.g. metabolite as different metabolites can be added to different wells at the same plate. Possible values: *metabolite, glucose, medium, starting PH, strain, volume*.
- *plate factor*, an experimental factor pertinent to a whole plate, e.g. *temperature, duration, reading interval*; it cannot be applied for a separate well.

Eve-CV provides definitions, possible and default values for the specified terms. For example, it defines the following for the term medium (see Fig. 1):

```
Term: Medium
hasSynonym: GrowthMedium
Definition: an experimental factor specifying a mixture for growing yeast culture.
Possible values: YNB (minimal media), YNBandAminoAcids, YPD (rich media)
Cardinality: 1
Default value: YPD
```

Figure 1: Example of a term defined in Eve-CV.

15.2. SciCom

We developed a dedicated communication mechanism SciCom (<u>Sci</u>entific <u>Com</u>munication) to support the proposed AdaLab framework, to communicate information about experiments between Paris and Manchester. SciCom defines a protocol for:

- requests of Eve experiments by AdaLab agents: human scientists, simulation software and machine learning algorithms.
- notifications by Eve to AdaLab agents about experimental results.

The semantics of these requests is defined by Eve-CV. It defines typical Eve experiments with corresponding experimental factors and outputs. Requests for experiments specify only the differences with a defined standard experiment. Namely, requests typically contain a list of yeast strains to experiment with, and outputs, other than OD (Optical Density) measurements.

SciCom has been implemented in YAML (/ˈjæməl/, rhymes with camel), a human-readable data serialization language[5]. It is commonly used for configuration files, but is suitable for many applications where data is being stored or transmitted. An example request for an experiment is shown in Figure 2.


```
%YAML 1.2
experiment id: 001
hasStandartPlateFactor:
- true
#  this means standard conditions for plate are:
# Oxygenation: LidLift
# Shaking: DoubleCircularShaking
# Reading Interval: 20
# Temperature: 25
# Duration: 48
# PlateFormat: 384

wellFactor:
Strains:
- WT (D273-10B)
- WT (BY4741)
# other factors are standard for all wells:
# Glucose: 0
# Medium: YPD
# Volume: 80
# StartingPH: 3

output:
- Glucose
- EtOH
# standard outputs - OD
... …
```


Figure 2: Example request for an experiment 001 in YAML.

An example of outputs of the experiment ID:001 is shown in Figure 3. Using ID, all output data for this experiment can be retrieved from the database.


```
%YAML 1.2
experiment id: 001
StandartODMeasurementExperiment:
- true
wellFactor:
Strain: [D273-10B, BY4741]
output:
- Glucose
- EtOH
ODSeries:
PlateID # plate barcode
WellID #A01 to P24
 [(TimeReadingPoint1,Value1),
(TimeReadingPoint2,Value2)..(TimeReadingPointN,ValueN)]
GlucoseSeries:
PlateID # plate barcode
[(TimeReadingPoint1,Value1),
(TimeReadingPoint2,Value2)..(TimeReadingPoint11,Value11)]
```


[5] http://www.yaml.org/spec/1.2/spec.html

```
AlcoholSeries:
PlateID # plate barcode
[(TimeReadingPoint1,Value1),
(TimeReadingPoint2,Value2)..(TimeReadingPoint11,Value11)]
```

Figure 3: Example output of the experiment 001 in YAML.

The experimental requests and results are stored in a RDF triple store in Manchester; it consists of 10,187,417 RDF triples combined in two graphs. Software agents can process the requests and notifications in YAML.

References

1. He, Y. and Xiang, Z. (2013). HINO: a BFO-aligned ontology representing human molecular interactions and pathways, CoRR abs/1311.3355.
2. Hilario, M.; Keet, M.; Lawrynowicz, A.; d'Amato null, C.; Do, H.; Fischer, S.; Gamberger, D.; Al-Jadir, L.; Jupp, S.; Kalousis, A.; Novak, P. K.; Mougouie, B.; Nguyen, P.; Palma, R.; Stevens, R.; Vavpetic, A.; Wang, J.; Wijaya, D. and Woznica, A. (2012). e-lico Data Mining Ontology (DMO) for Data Mining Optimization (DMOP).
3. Ghazvinian, A., Noy, N. F. and Musen, M. A. (2011). How orthogonal are the OBO Foundry ontologies?, J. Biomedical Semantics 2 : S2.

16. Scalability

Different parts of the AdaLab pipeline have different sensitivity to the increase in the input network size.

Learning/revising a regulation network: CoRegNet, that relies on the h-LICORN algorithm (Chebil *et al.*, 2014; Elati *et al.*, 2007) has been successfully applied to human datasets (Chebil *et al.*, 2014). As CoRegNet allows selecting relevant network nodes, its scalability is important to the remainder of the process. Concerning the regulation network, application to a mammalian system would mean dealing with a network with a larger number of nodes, and also an increased average |regulators| per node ratio.

The ELSA strategy used to extend Mz is of quadratic complexity with the number of nodes, and of linear complexity with the number of learned models, which makes it suitable for dealing with bigger regulatory graphs. The learning of multiple models is highly parallelizable, making it tailored for computation on distributed architectures.

The MinerLC graph mining algorithm was applied in this study to a co-regulation network with less than 200 nodes described by a language of 21 labels describing influence profiles. This algorithm has also been tested on several networks of different nature and size (up to 4 orders of magnitude larger in size). Within the limit of two orders of magnitude in number of vertices and edges and for a number of labels of similar size the execution times remain reasonable (<10s), and it is not necessary to introduce any particular constraints to limit its outputs. On this basis, assuming that the protocol remains similar (same labelling method), we argue that the increase in network size resulting from the transition from yeast to mammals should not be a problem.

Withg less studied organisms, the *a priori* network might be missing or very sparse. In such a case, combining CoRegNet and ELSA would allow learning a network from scratch (gene expression datasets only) to bootstrap the revision process.

AdactiveFB relies on multiple operational steps. For every real experiment data available, both a forward and a backward simulation must be performed on the current model. Forward simulation alternates for the number of experiment time points between the dynamic FBA computation and the DBN inference steps, dominated by the former, i.e. a LP constrained by a stoichiometric matrix which size depends on the number of metabolite and reactions in the metabolic model. Backward simulation first requires a pre-processing phase, where a real experiment growth curve is reversed to a sequence of partial metabolic gene states (one state for each time point) by transforming the dFBA objective function to match the actually observed growth value at each time point and finding the corresponding flux values to then partially infer metabolic gene values. The computation in the pre-processing step is also dominated by the dFBA steps, and can be cached since it does not depend on the current model. Backward simulation for a current model then only considers DBN backward inference, which easily scales to larger networks. The final steps of AdactiveFB are dominated by the distance computation between each (experiment, time, gene) triple between forward and backward simulation inferred marginal distributions, and are thus linear with respect to each of the triple components, allowing to scale to bigger networks.

References

Chebil, I., Nicolle, R., Santini, G., Rouveirol, C., & Elati, M. (2014). Hybrid method inference for the construction of cooperative regulatory network in human. *NanoBioscience, IEEE Transactions on, 13*(2), 97-103.

Elati M, et al. (2007) LICORN: learning cooperative regulation networks from gene expression data. Bioinformatics. 2007;23:2407–2414. doi: 10.1093/bioinformatics/btm352.
